# RNA Sequencing Reveals the Upregulation of FOXO Signaling Pathway in *Porphyromonas gingivalis* Persister-Treated Human Gingival Epithelial Cells

**DOI:** 10.3390/ijms23105728

**Published:** 2022-05-20

**Authors:** Chuan Wang, Xuan Li, Tianfan Cheng, Leilei Wang, Lijian Jin

**Affiliations:** Faculty of Dentistry, The University of Hong Kong, Hong Kong SAR, China; chuan525@connect.hku.hk (C.W.); lixuanlwj@hotmail.com (X.L.); chengtfc@hku.hk (T.C.); lei0302@connect.hku.hk (L.W.)

**Keywords:** *Porphyromonas gingivalis* persisters, human gingival epithelial cells, RNA sequencing, innate host defense, FOXO signaling pathway

## Abstract

*Porphyromonas gingivalis* as the keystone periodontopathogen plays a critical role in the pathogenesis of periodontitis, and crucially accounts for inflammatory comorbidities such as cardiovascular disease and Alzheimer′s disease. We recently identified the existence of *P. gingivalis* persisters and revealed the unforeseen perturbation of innate response in human gingival epithelial cells (HGECs) due to these noxious persisters. Herein, RNA sequencing revealed how *P. gingivalis* persisters affected the expression profile of cytokine genes and related signaling pathways in HGECs. Results showed that metronidazole-treated *P. gingivalis* persisters (M-PgPs) impaired the innate host defense of HGECs, in a similar fashion to *P. gingivalis*. Notably, over one thousand differentially expressed genes were identified in HGECs treated with M-PgPs or *P. gingivalis* with reference to the controls. Gene Ontology and KEGG pathway analysis demonstrated significantly enriched signaling pathways, such as FOXO. Importantly, the FOXO1 inhibitor rescued the M-PgP-induced disruption of cytokine expression. This study suggests that *P. gingivalis* persisters may perturb innate host defense, through the upregulation of the FOXO signaling pathway. Thus, the current findings could contribute to developing new approaches to tackling *P. gingivalis* persisters for the effective control of periodontitis and *P. gingivalis*-related inflammatory comorbidities.

## 1. Introduction

Periodontal diseases (gingivitis and periodontitis) are among the major global oral health burdens, with disastrous socioeconomic impacts and humongous healthcare costs [1,2,3,4,5], and yet periodontitis is intimately linked to over 50 systemic diseases and conditions, such as diabetes mellitus, cardiovascular disease, cancer and Alzheimer′s disease [6,7,8,9]. Severe periodontitis, affecting 11.2% of the entire global population, was ranked as the 6th most prevalent disease in humans in the first Global Burden of Disease (GBD) study [4,5,8,10]. If uncontrolled, it may eventually lead to severe tooth loss and edentulous in adults. *Porphyromonas gingivalis*, the major periodontopathogen, has been recognized as the ‘keystone’ pathogen for periodontitis [11]. It enables the disturbance of microbe–host homeostasis by shifting microbial symbiosis/eubiosis to dysbiosis, even at a low abundance, leading to dysregulated immuno-inflammatory response and irreversible periodontal destruction [12,13]. Increasing evidence has linked periodontitis with other inflammatory comorbidities, and indeed, *P. gingivalis* plays multiple essential roles in this process [14,15]. Currently, it remains a challenge in clinical practice to precisely eliminate *P. gingivalis* cells in their biofilm and intracellular modes. This ‘keystone’ periodontopathogen develops tough intrinsic strategies to disrupt host immunity and survive under harsh environments [16,17], notably by forming persister cells as one of the key approaches [18,19].

Microbial persisters are a unique subpopulation of microorganisms with specific strategies to survive under the strain of a lethal dosage from multiple antimicrobials [20]. The first description of persisters can be dated back to 1944 [21] when Joseph W. Bigger found that penicillin failed to eliminate all of the cultured *Staphylococcus*. In fact, the remaining survivors, so-called ‘persisters’, are present in a dormant and non-dividing state, like bacteria in their stationary phase. After being overlooked for a half-century, there has been a rapid increase in interest and investigations on persisters over the past two decades. To date, persisters have been reported in almost all microorganisms tested [22], and several clinical symptoms, such as cystic fibrosis [23], oral carriage [24], urinary tract infections [25] and tuberculosis [26], have been closely associated with persisters. It is worth noting that it has been demonstrated that *Salmonella* persisters can dampen innate immune response and induce anti-inflammatory macrophage polarization, as well as enhance antibiotic resistance in the intestine [27,28]. Given the essential role that *P. gingivalis* plays in the relapse of periodontitis, our group has provided the first evidence of the presence of metronidazole-tolerant *P. gingivalis* persister cells [18], and further demonstrated that these metronidazole-treated *P. gingivalis* persisters (M-PgPs) enabled the suppression of the immuno-inflammatory response in human gingival epithelial cells (HGECs) [19], whereas the underlying mechanisms and related signaling pathways need further investigation.

HGECs as the frontline defense of periodontal tissues function as both physical [29] and biological barriers for maintaining periodontal homeostasis and health [30]. When challenged by pathogenic bacteria, HGECs are able to secrete pro-inflammatory cytokines and chemokines, such as C-X-C motif chemokine 5 (CXCL5), Interleukin (IL)-6 and IL-8 [31,32], which exhibit the potent effects on the recruitment of neutrophils [33], thereby playing a critical role in maintaining periodontal health. The inhibitory effects of M-PgPs on the expression of CXCL5, IL-6 and IL-8 demonstrated by our group show that even when treated with a lethal concentration of antibiotics, M-PgPs still disrupt the innate host responses of HGECs. Thus, targeting this inhibitory activity of M-PgPs could serve as a potential strategy to control periodontitis and *P. gingivalis*-related inflammatory comorbidities.

Here, the present study further investigated how these M-PgPs interacted with HGECs and affected the innate host defense. It was hypothesized that certain signaling pathways could be critically involved in the M-PgP-induced disruption of immuno-inflammatory responses in HGECs, and thus, this RNA sequencing (RNA-seq) study aimed to identify candidate genes and underlying signaling pathways.

## 2. Results

### 2.1. M-PgPs Inhibited the Innate Host Defense in HGECs

**Figure 1:**
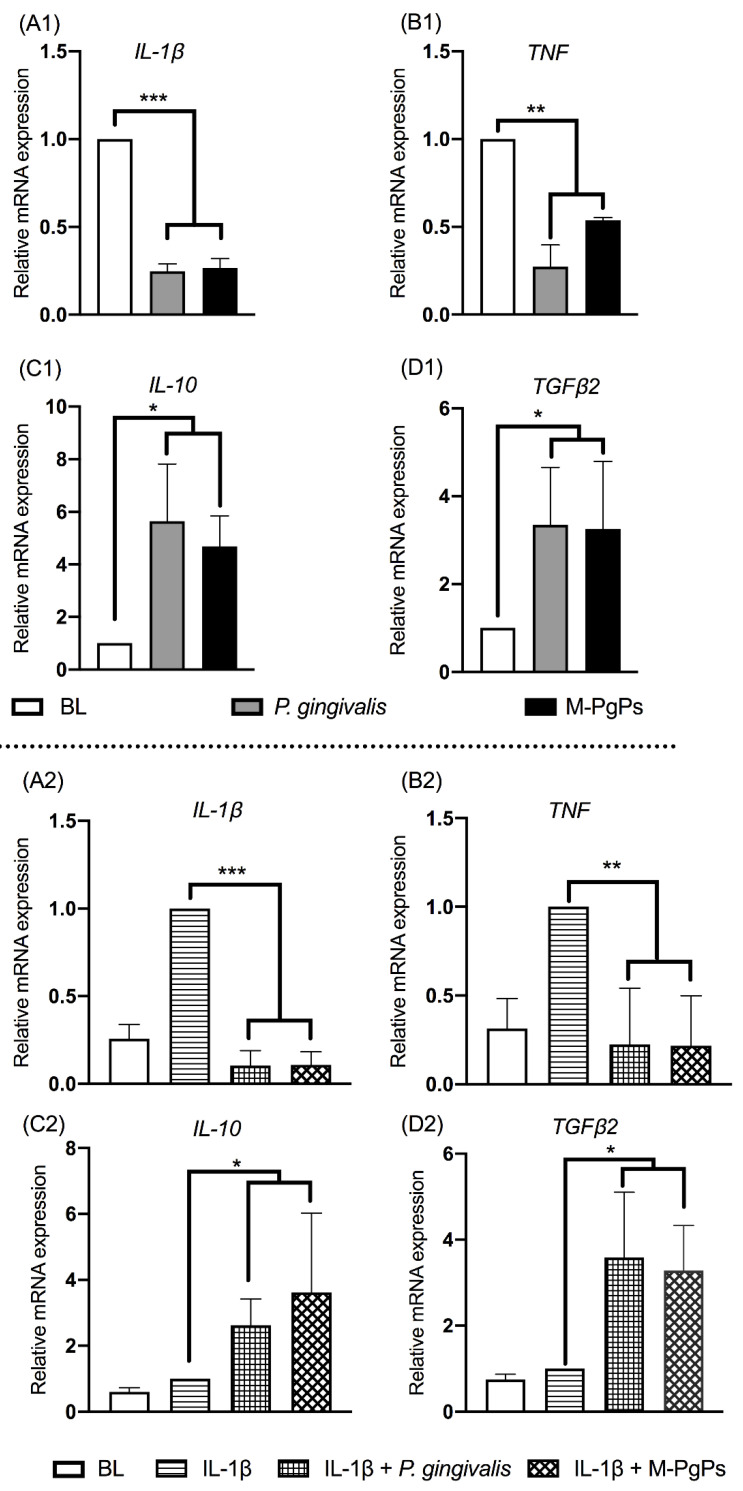
M-PgPs inhibit the innate host defense of HGECs. Relative mRNA expression levels of *IL-1β* (**A1**,**A2**), *TNF* (**B1**,**B2**), *IL-10* (**C1**,**C2**), and *TGF-β2* (**D1**,**D2**)in HGECs after the treatment of M-PgPs (1:100) for 24 h were analyzed using RT-qPCR. (**A1**–**D1**) Under normal condition without IL-1β pre-treatment. (**A2**–**D2**) Under inflammatory condition with IL-1β pre-treatment. HGECs without any treatment served as blank control (BL). HGECs treated with *P. gingivalis* (1:100, 24 h) served as positive control. IL-1β (1 ng mL^−1^) was added 6 h before M-PgPs and *P. gingivalis* treatment. * *p* < 0.05; ** *p* < 0.01; *** *p* < 0.001. Data represent the mean ± SD of three independent experiments.

The previous study of our group showed that cytokine (IL-6, IL-8, and CXCL5) expression levels in HGECs were remarkably suppressed by M-PgPs [19]. Herein, we further investigated the relative mRNA expression levels of pro-inflammatory (*IL-1β* and *TNF*) and anti-inflammatory cytokines (*IL-10* and *TGF-β2*) in M-PgP-treated HGECs. IL-1β (1 ng mL^−1^) was used for the pretreatment of HGECs to mimic the inflammatory condition. Notably, *IL-1β* and *TNF* were downregulated, while *IL-10* and *TGF-β2* were upregulated by M-PgPs, with reference to the untreated group, whether they were pre-treated with IL-1β or not (Figure 1). These findings showed that M-PgPs enabled the inhibition of the innate host defense in HGECs.

### 2.2. Differential Gene Expression

To verify the underlying mechanisms behind the inhibitory effects of M-PgPs on the immuno-inflammatory response of HGECs, RNA-seq was conducted to determine the possible genes and signaling pathways that could be involved in this process. Samples from six groups were compared: HGECs only (blank group, BL), M-PgP-treated HGECs, *P. gingivalis*-treated HGECs, IL-1β-treated HGECs (negative control group, NC), IL-1β + M-PgP-treated HGECs and IL-1β + *P. gingivalis*-treated HGECs. After sequencing, a differentially expressed gene (DEG) analysis was performed to identify gene expression changes among these groups.

Under the normal condition without IL-1β pretreatment, 1237 DEGs (*p* < 0.05, |Log2 fold change| ≥ 1) were identified in M-PgP-treated HGECs as compared with BL (Figure S1A), including 689 upregulated genes (Figure S2A) and 548 downregulated ones (Figure S2B). Moreover, there were totally 1452 DEGs in *P. gingivalis*-treated HGECs with reference to BL (Figure S1B), including 778 upregulated genes (Figure S2A) and 674 downregulated genes (Figure S2B). Under inflammatory condition with IL-1β pretreatment, a total of 1135 DEGs were identified in IL-1β + M-PgP-treated HGECs compared with NC (Figure S1C), which included 724 upregulated genes (Figure S2A) and 411 downregulated genes (Figure S2B). Furthermore, there were totally 1393 DEGs in IL-1β + *P. gingivalis*-treated HGECs referred to NC (Figure S1D), including 876 upregulated genes (Figure S2A) and 517 downregulated ones (Figure S2B).

Notably, some genes were similarly identified as DEGs in all groups, among which the top 20 DEGs are shown in Table S1. Importantly, no DEGs were identified between M-PgP- and *P. gingivalis*-treated HGECs, whether they were pre-treated with IL-1β or not.

### 2.3. Gene Ontology (GO) Functional Annotation and Enrichment Analyses

**Figure 2:**
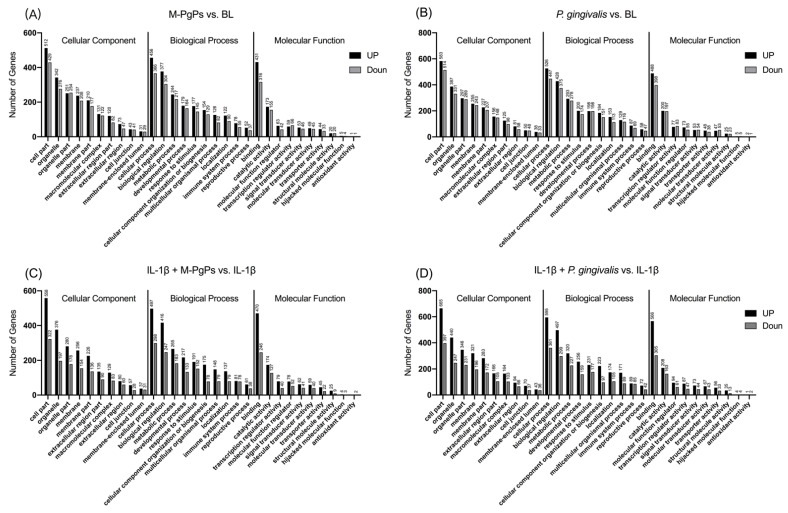
Gene Ontology functional annotation of DEGs in different comparison groups. The top 10 terms of GO functional annotation assigned from the DEGs in different comparison groups are shown. M-PgPs vs. BL (**A**); *P. gingivalis* vs. BL (**B**); IL-1β + M-PgPs vs. IL-1β (**C**); and IL-1β + *P. gingivalis* vs. IL-1β (**D**). GO terms are shown on the x axis. Numbers of DEGs enriched in the terms are shown above the bar. Black bar: upregulated genes. Gray bar: downregulated genes. Samples for RNA-Seq were obtained from three independent experiments.

To generally describe the functions of the DEGs obtained from RNA-Seq, functional annotation was performed by comparing the sequences with the GO databases. DEGs annotated by the GO databases were then classified into three categories, including the cellular component (CC), biological progress (BP) and molecular function (MF). In total, 52 terms were assigned from the DEGs, and the top 10 terms of each category based on the number of DEGs assigned are shown in Figure 2.

Enrichment analyses of GO terms were performed for DEGs using GOatools. GO terms with q < 0.05 were identified as significantly enriched. According to the functional enrichment results, several terms were significantly enriched within each category (CC, BP and MF) (Table S2). The top 20 terms of each category based on the Rich Ratio are shown in Table S3.

### 2.4. KEGG Pathway Enrichment Analysis

**Figure 3:**
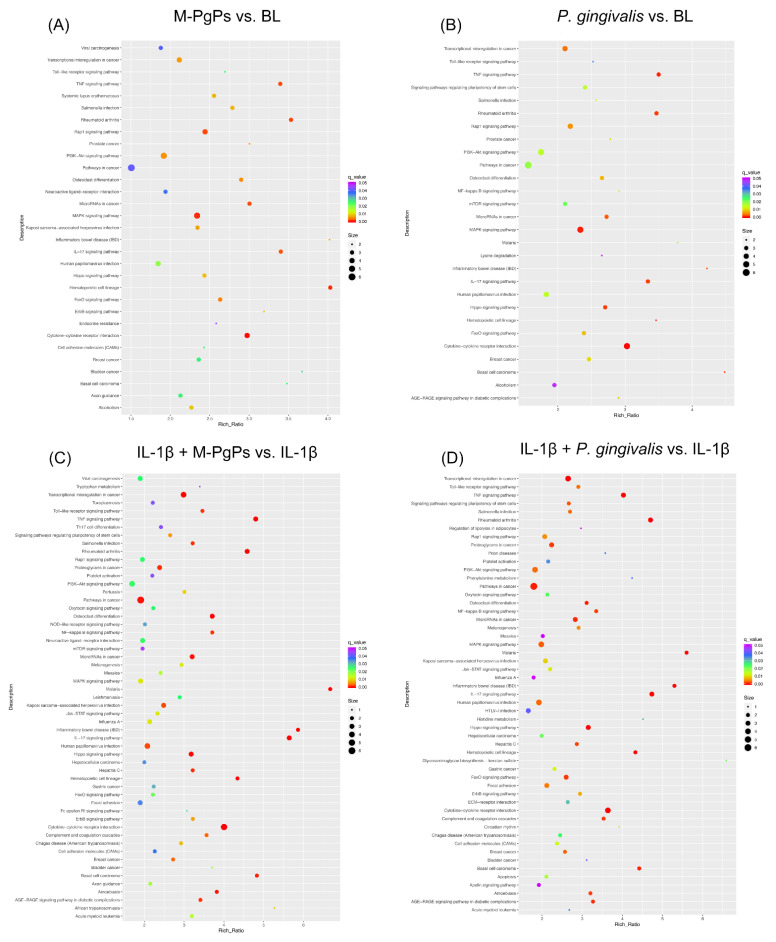
KEGG pathway enrichment analysis of DEGs in different comparison groups. M-PgPs vs. BL (**A**); *P. gingivalis* vs. BL (**B**); IL-1β + M-PgPs vs. IL-1β (**C**); and IL-1β + *P. gingivalis* vs. IL-1β (**D**). Rich Ratio is shown on the x axis. KEGG pathway names are shown on the y axis. Size of the dots represents numbers of genes enriched in the term. Color of the dots represents q value. Samples for RNA-Seq were obtained from three independent experiments.

This analysis was performed to explore the enriched pathways for DEGs. As compared to the BL group, 31 and 28 KEGG pathways were identified as significantly enriched in the M-PgP-treated and *P. gingivalis*-treated HGECs, respectively (q < 0.05). It is worth noting that the most enriched pathway in both groups was Cytokine-cytokine receptor interaction. Additionally, the specific pathways of MAPK, TNF and FOXO were also significantly enriched in both groups (Figure 3A,B).

With reference to the NC group, 55 and 53 KEGG pathways were identified as significantly enriched in the IL-1β + M-PgP-treated and IL-1β + *P. gingivalis*-treated HGECs, respectively (q < 0.05). The most enriched pathway in both groups was the cytokine–cytokine receptor interaction. Additionally, TNF and IL-17 signaling pathways, as well as pathways in cancer and transcriptional misregulation in cancer, were also significantly enriched in both groups (Figure 3C, D).

Several signaling pathways (e.g., cytokine–cytokine receptor interaction, TNF signaling, IL-17 signaling, FOXO signaling, pathways in cancer, PI3K-Akt signaling and Toll-like receptor signaling) were identified as significantly enriched in all comparison groups (Figure 3). The numbers of genes mapped onto these signaling pathways are shown in Table S4.

### 2.5. FOXO Signaling Pathway Was Involved in the Inhibitory Effects of M-PgPs on the Innate Host Defense in HGECs

**Figure 4:**
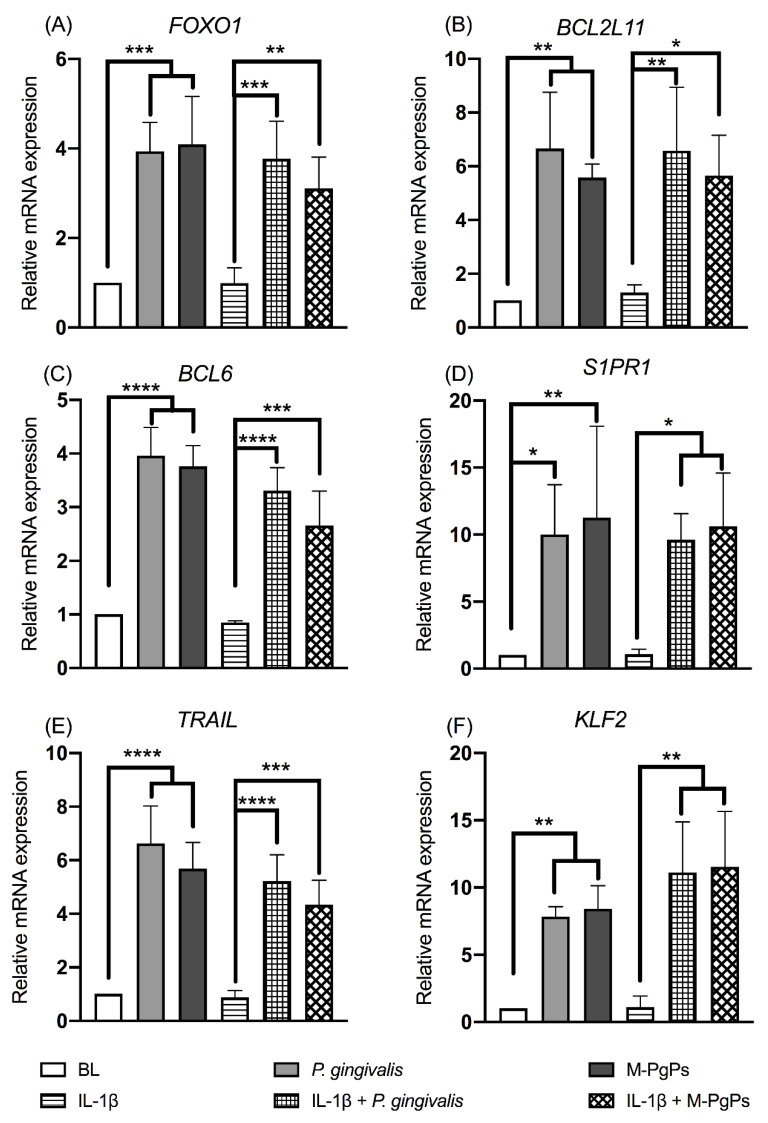
FOXO signaling pathway in HGECs was upregulated by M-PgPs. Relative mRNA expression levels of *FOXO1* (**A**), *BCL2L11* (**B**), *BCL6* (**C**), *S1PR1* (**D**), *TRAIL* (**E**) and *KLF2* (**F**) in HGECs after the treatment of M-PgPs (1:100) for 24 h were analyzed using RT-qPCR. HGECs without any treatment served as blank control (BL). HGECs treated with *P. gingivalis* (1:100, 24 h) served as positive control. IL-1β (1 ng mL^−1^) was added 6 h before M-PgPs and *P. gingivalis* treatment. * *p* < 0.05; ** *p* < 0.01; *** *p* < 0.001; **** *p* < 0.0001. Data represent the mean ± SD of three independent experiments.

Since the FOXO signaling pathway was identified as significantly enriched in all groups, further verification assay was conducted. The relative mRNA expression levels of *FOXO1* and several downstream genes (e.g., *BCL2L11*, *BCL6*, *S1PR1*, *TRAIL* and *KLF2*) were analyzed using RT-qPCR, thereby demonstrating that these gene expression levels were upregulated in both *P. gingivalis*- and M-PgP-treated HGECs as compared to the BL/NC groups (Figure 4). Then, the FOXO1 inhibitor (Fi) was used to block the FOXO signaling pathway. The preliminary results showed that the most appropriate concentration of Fi was 1 μM, and it could downregulate the relative mRNA expression levels of *FOXO1*, *KLF2* and *TRAIL* (Figure S3). It is worth noting that higher Fi concentrations (≥2 μM) could lead to the death of HGECs, resulting in a cell viability of lower than 80% (Figure S3).

**Figure 5:**
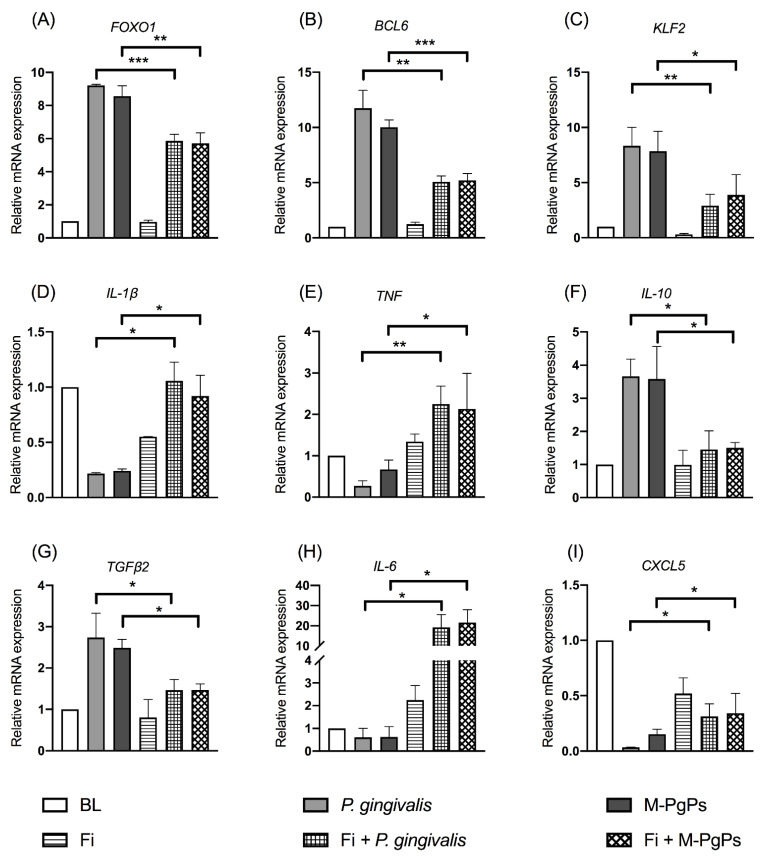
FOXO1 inhibitor (Fi) rescued the inhibitory effects of M-PgPs on the immuno-inflammatory response of HGECs under normal condition. Relative mRNA expression levels of *FOXO1* (**A**), *BCL6* (**B**), *KLF2* (**C**), *IL-1β* (**D**), *TNF* (**E**), *IL-10* (**F**), *TGF-β2* (**G**), IL-6 (**H**) and *CXCL5* (**I**) in HGECs after the treatment of M-PgPs (1:100) for 24 h were analyzed using RT-qPCR. HGECs without any treatment served as blank control (BL). HGECs treated with *P. gingivalis* (1:100, 24 h) served as positive control. Fi (1 μM) was added 2 h before M-PgPs and *P. gingivalis* treatment. * *p* < 0.05; ** *p* < 0.01; *** *p* < 0.001. Data represent the mean ± SD of three independent experiments.

Under the normal condition, Fi reversed the relative expression levels of *FOXO1* mRNAs and the downstream genes (*BCL6* and *KLF2*) affected by M-PgPs (Figure 5A–C). In parallel, the downregulated *IL-1β* and *TNF* as well as the upregulated *IL-10* and *TGFβ2* due to M-PgPs were completely or partly reversed by Fi (Figure 5D–G). Moreover, the downregulated cytokines (*IL-6* and *CXCL5*) were also fully or partly reversed by Fi (Figure 5H and I), whereas under the inflammatory condition, Fi did not affect the *FOXO1* mRNA expression level (Figure S4A) but reversed downstream genes such as *KLF2* (Figure S4B). Meanwhile, the altered mRNA expression levels of *IL-1β*, *TNF*, *IL-10*, *TGFβ2*, *IL-6*, *IL-8* and *CXCL5* were partly reversed by Fi (Figure S4C–I).

## 3. Discussion

In the present study, RNA-seq analysis was undertaken to identify the potential mechanisms (e.g., the candidate genes and signaling pathways) underlying the *P. gingivalis* persister-induced disruption of immuno-inflammatory responses in HGECs. The findings indicated that the FOXO signaling pathway was crucially involved in this process. Interestingly, the rescue experiment verified that the FOXO1 inhibitor could reverse the cytokine expression levels altered by M-PgPs. Our current work demonstrates that M-PgPs can impair innate host defense through the upregulation of the FOXO signaling pathway. This study may contribute to developing novel approaches to tackling *P. gingivalis* persisters for effectively controlling periodontitis and *P. gingivalis-*related inflammatory comorbidities.

As the first line of defense in the periodontium, gingival epithelial cells play essential roles in maintaining periodontal homeostasis and health. Thus, numerous studies have investigated the immuno-inflammatory responses in HGECs challenged by pathogenic bacteria such as *P. gingivalis* [34,35,36]. Our group has recently demonstrated, for the first time, that M-PgPs perturb the innate immune responses in HGECs [19] in an identical fashion to *P. gingivalis*. Similar results were found in the present study, showing that the pro-inflammatory cytokine genes (*IL-1β* and *TNF*) were downregulated, while the anti-inflammatory cytokine genes (*IL-10* and *TGF-β2*) were upregulated by M-PgPs, with reference to the untreated group. However, the underlying mechanisms involved in the M-PgP-induced disruption of innate host defense and the relevant biological implications remain unclear.

In this study, RNA sequencing was performed in HGECs after treatments of M-PgPs and *P. gingivalis*, and over one thousand DEGs were identified among the groups. Interestingly, no notable inter-group difference was identified under both normal and inflammatory conditions. This finding indicates that M-PgPs maintain a similar capability to *P. gingivalis* in suppressing immuno-inflammatory responses in HGECs in accordance with our previous study [19]. Further study is required to confirm this point, and explore the molecular signature of *P. gingivalis* persister pathogenicity.

Next, GO term assignment and enrichment analyses showed that multiple GO terms were significantly enriched in all groups (e.g., cell junction, metabolic process, response to stimulus, transcription regulator activity, translation regulator activity and antioxidant activity). These annotations and classifications provide us with plenty of resources and information for illustrating the underlying processes, functions and pathways related to the actions of HGECs in response to certain pathogens and their persisters.

Moreover, KEGG pathway enrichment analyses demonstrated that the FOXO signaling pathway was significantly enriched in M-PgP-treated HGECs. It is well known that the FOXO transcription family consists of four members (FOXO1, FOXO3, FOXO4 and FOXO6) in mammals and acts as a critical signaling mediator in various cell biological processes, such as oxidative stress responses, apoptosis, proliferation, energy metabolism and inflammation [37]. Particularly, FOXO plays critical roles in the homeostasis of immune-relevant cells [38,39]. As the representative and the best studied member of the FOXO family [40], FOXO1 has been investigated in various cell types and genetically modified mice models [41,42,43,44], while only a few studies have explored its functions in HGECs. Further investigation on the modulation of FOXO in HGECs would enable us to gain a better understanding of the initiation and progression of periodontitis. Our study revealed that the mRNA levels of *FOXO1* and its downstream genes were upregulated by M-PgPs and *P. gingivalis*. This finding is consistent with a previous study in which *P. gingivalis* could induce the activation of FOXO1 in gingival epithelial cells [45]. Considering the finding that M-PgPs suppressed the immuno-inflammatory response in HGECs, the FOXO signaling pathway may be involved in this notable observation.

In order to confirm this hypothesis, FOXO1 inhibitor (Fi) was applied, as previously described [46], to perturb activation by M-PgPs. It is worth noting that Fi totally or partly rescued the relative mRNA expression levels of those genes affected by M-PgPs under normal and inflammatory conditions. Thus, M-PgPs could perturb the innate response of HGECs, at least partly, through the upregulation of the FOXO signaling pathway, and blocking the FOXO signaling pathway may possibly disrupt this process.

The Fi (AS1842856) was first discovered by Nagashima and co-workers for treating type 2 diabetes mellitus [46], and indeed, it has been widely used for the management of diabetes in animal models [47,48,49]. In the present study, Fi was used to treat HGECs for the first time, and surprisingly, it could to some extent rescue the M-PgP-induced disruption of immuno-inflammatory responses. Notably, Fi is a specific and powerful inhibitor of FOXO1, and the current finding confirms the essential regulatory effects of the FOXO signaling pathway in the M-PgP-dysregulated innate response in HGECs. Further studies are warranted to determine the potential usage of Fi in other FOXO-related research in connection to the pathogenicity of *P. gingivalis* [45] and M-PgPs.

Besides FOXO, this study also identified other signaling pathways that were highly enriched in all groups, such as cytokine–cytokine receptor interaction, the TNF signaling pathway and the IL-17 signaling pathway. Cytokines as a broad category of small proteins play essential roles in immunoregulatory and inflammatory processes. The resultant actions need to be exerted through the interaction with specific receptors. Thus, these cytokine-related signaling pathways critically account for microbe-induced inflammatory responses. Remarkably, the majority of the DEGs enriched in these pathways (e.g., *TNF*, *CCL2*, *CCL20*, *FOS*, *IL-1* and *TNFAIP3*) revealed a downregulated trend, which is in accordance with the observation of the inhibition of the innate host defense to M-PgPs and *P. gingivalis*. It is noted that multiple signaling pathways could be involved in this process. Thus, further investigations are needed to better illustrate the regulatory mechanisms and develop more effective solutions.

It is worth noting that *P. gingivalis* persisters can maintain their pathogenicity even when treated with a lethal concentration of metronidazole [19]. This finding inspires us to reconsider the rationale and strategy of antibiotic usage in clinical practice. More studies are required to develop novel and precise approaches to tackling microbe-induced inflammatory diseases and systemic comorbidities. Indeed, we have recently demonstrated that bismuth drugs can rescue *P. gingivalis*-perturbed innate host responses [50], and *P. gingivalis* persisters could be effectively eliminated via the synergistic combination of bismuth drugs such as colloidal bismuth subcitrate (CBS) with metronidazole [51]. Further studies are required to develop new anti-persister drug delivery systems to effectively tackle bacterial persisters and modulate dysregulated immunoinflammatory responses to control *P. gingivalis*-related diseases.

Nevertheless, there are several limitations of this study. For instance, persister cells only take up a small proportion of the whole population, and it remains challenging to precisely isolate them from unlysed cells after antimicrobial treatments [52,53]. As such, in our previous work [19] and this study, we used the term ‘metronidazole-treated *P. gingivalis* persisters (M-PgPs)’, which reflected the components of *P. gingivalis* persisters and metronidazole-killed *P. gingivalis* cells. Moreover, we only verified the RNA-Seq results using RT-qPCR, but did not assess the protein expression levels. It is noted that protein expression is a complicated process, and it is affected by different microenvironmental conditions. Indeed, proinflammatory cytokines could be possibly degraded by *P. gingivalis* gingipains [54,55]. Further investigation must extend to FOXO transcription factors and proteins to clarify these points.

## 4. Materials and Methods

### 4.1. Culture of Cells

HGECs obtained from CELLnTEC (CELLnTEC, Berne, Switzerland) were used in this study as our established protocol [19]. They were cultured in epithelial culture medium (CnT-prime, CELLnTEC, Berne, Switzerland, changed every two days) in a humidified incubator (37 °C with 5% CO_2_). The 3rd to 5th passages of HGECs were used in all experiments.

### 4.2. Bacterial Culture and M-PgPs Formation

*P. gingivalis* (ATCC 33277) was employed and cultured following our established protocol [19]. Frozen stocked bacteria were first grown on blood agar plates (44 g L^−1^ BD Columbia agar base (Becton Dickinson GmbH, Heidelberg, Germany), 5% horse blood (Hemostat, Dixon, CA, USA), 5 mg L^−1^ hemin (Sigma-Aldrich, St. Louis, MO, USA), 1 mg L^−1^ vitamin K1 (Sigma-Aldrich, St. Louis, MO, USA)) in an anaerobic atmosphere at 37 °C (10% H_2_, 5% CO_2_ and 85% N_2_). After one week, a single colony was picked and placed in liquid Trypticase soy broth (30 g L^−1^ TSB; Becton Dickinson GmbH, Heidelberg, Germany) supplemented with 5 g L^−1^ yeast extract, 5 mg L^−1^ hemin and 1 mg L^−1^ vitamin K1; it was then cultured under the same anaerobic conditions.

M-PgPs formation was performed following our previous study [19]. In brief, *P. gingivalis* was cultured in broth for 48 h and re-suspended in fresh broth to an OD600 of 0.1. Subsequently, the bacteria were incubated in the stationary phase (72 h) and further treated with metronidazole (MTZ, Sigma-Aldrich, St. Louis, MO, USA) at 100 mg L^−1^ for 6 h. It was confirmed by our recent study that about 1% of the *P. gingivalis* cells remained viable after the 6 h MTZ treatment [51].

### 4.3. Infection of HGECs with M-PgPs

HGECs (4 × 10^5^ cells) were seeded into 6-well plates. Following cellular adherence, the cells were pre-treated with or without IL-1β (1 ng ml^−1^) to mimick the inflammatory condition. Those without IL-1β treatment were defined as the blank control (BL). After 4 h, cells were treated with 1 μM FOXO1 inhibitor (Fi) AS1842856 [46] (Selleckchem, Houston, TX, USA) or not. After another 2 h, cells were infected with M-PgPs or *P. gingivalis* (MOI: 100) for 24 h, according to previous studies [19,56,57]. Total RNAs were collected for the following tests.

### 4.4. RT-qPCR

Total RNA (600 ng) was used to synthesize cDNA with the QuantiTect Reverse Transcription Kit (Qiagen, Benelux BV Qiagen GmbH, Hilden, Germany). RT-qPCR was undertaken using the ABI 7500 Real-time PCR System (Applied Biosystems, Carlsbad, CA, USA) with the QuantiNova SYBR Green PCR Kit (Qiagen, Benelux BV Qiagen GmbH, Hilden, Germany). The expression level of individual genes was normalized to β-actin using the comparative 2^−ΔΔCT^ method. The primer sequences are listed in Table 1.

### 4.5. Library Preparation and RNA Sequencing

Library preparation, RNA-seq and data analysis were performed at the Annoroad Gene Technology Corporation (Beijing, China). The samples were obtained from three independent experiments. Total RNA was purified using the RNeasy Plus Mini Kit (Qiagen, Benelux BV Qiagen GmbH, Hilden, Germany). Annoroad Gene Technology Corporation (Beijing, China) constructed cDNA libraries and performed sequencing using these RNA samples. In brief, after RNA quantity and quality detection and RNA fragment size analysis, mRNA was enriched from total RNA using oligo (dT) magnetic beads. Then, sequencing libraries were prepared using the NEBNext^®^ Ultra™ RNA Library Prep Kit for Illumina^®^ (#E7530L, New England Biolabs, Inc, Ipswich, MA, USA), and index codes were added for attribute sequencing. The library RNA concentration was measured using the Qubit^®^ RNA Assay Kit (Life technologies, Waltham, MA, USA) for initial quantification, and then adjusted to 1 ng μL^−1^. To make sure the insert size matched the valid library concentration (>10 nM), it was tested and accurately quantified using the Bioanalyzer 2100 system (Agilent Technologies, Santa Clara, CA, USA) and the CFX96 RT-PCR System (Bio-Rad Laboratories, Hercules, CA, USA), respectively. The index-coded samples were clustered in a cBot cluster generation system using the HiSeq PE Cluster Kit v4-cBot-HS (Illumina, Santiago, CA, USA). Library sequencing was then undertaken using the NovaSeq 6000 platform (Illumina, Santiago, CA, USA) to generate 150 bp paired-end reads.

### 4.6. Data Filtering and Alignment

Raw reads generated from sequencing were filtered using Perl scripts [58] to (1) discard the reads if the length of the trimmed reads was lower than 30 bp after trimming the Smart-seq2 public primer sequence of the reads; (2) the contaminated reads were removed from adapters when the read bases contained more than 5 bp of adapter sequences; (3) the low-quality reads (carrying over 15% bases with a quality value ≤19) were removed; (4) reads that contained over 5% ambiguous nucleotides were removed. Following filtering, the clean reads were aligned to the reference genome (GRCh38.p13) using HISAT2 v2.1.0 (Baltimore, MD, USA) [59]. Herein, the reference and annotation files were obtained from the ENSEMBL browser (http://www.ensembl.org/index.html, accessed on 17 May 2022). A genome index was created using Bowtie2 v2.2.3 [60]. The multi-mapped and/or unmapped reads were excluded from the analysis.

### 4.7. Analysis of Differentially Expressed Genes

The transcript expression levels were determined using FPKM (Fragments per Kilobase per Million Mapped Fragments) with HTSeq v0.6.0 (California Institute of Technology, Pasadena, CA, USA) [61]. Correlation analysis was performed using the genes expressed in at least one sample. Hierarchical clustering was carried out via the Pearson correlation distance, and the expression levels of each gene in each sample were estimated with DESeq2 using linear regression. A *p*-value was calculated using the Wald test [62] and corrected using the Benjamini–Hochberg q-value (false discovery rate, FDR) [63]. The genes (q < 0.05 and |log2 ratio| ≥ 1) were then defined as differentially expressed genes (DEGs) [64].

### 4.8. Functional Annotation and Enrichment Analysis

Both GO (Gene Ontology, http://geneontology.org/, accessed on 17 May 2022) and KEGG (Kyoto Encyclopedia of Genes and Genomes, http://www.kegg.jp/, accessed on 17 May 2022) enrichments of DEGs were applied using the hypergeometric test. Fisher’s exact test and multiple comparisons were then performed to adjust the *p*-value as the q-value (false discovery rate, FDR). The genes in the whole genome served as the background of the datasets. They were determined to be significantly enriched, with q < 0.05 for the GO terms and KEGG pathways.

### 4.9. Cell Viability Test

HGECs (5 × 10^3^ cells) were seeded into 96-well plates. After adhesion, cells were treated with different concentrations of FOXO1 inhibitor (Fi) AS1842856 (0 μM to 100 μM) for 24 h. The cell viability was evaluated using a testing kit (CyQUANT™ MTT Cell Proliferation Assay, Thermo Fisher Scientific Inc., Waltham, MA, USA).

### 4.10. Statistical Analysis

All results were presented as the mean ± standard deviation (SD). At least 3 independent repeats were conducted separately in each experiment. Statistical calculations were performed using GraphPad Prism 8. Inter-group differences were determined via one-way analysis of variance. *p* < 0.05 was considered as statistically significant.

## 5. Conclusions

Our current study demonstrates significant DEGs in M-PgP-treated HGECs. Functional enrichment analyses of the DEGs using GO and KEGG identified greatly enriched terms and pathways involved in the M-PgP-induced inhibition of innate host defense in HGECs. Further verification confirmed that the FOXO signaling pathway may be involved in this observed action. These findings enhance our understanding of the specific survival strategies of M-PgPs after invading host cells. Targeting the FOXO signaling pathway could be an alternative approach to tackling *P. gingivalis* persisters for the effective control of periodontitis and *P. gingivalis*-related inflammatory comorbidities.

## Figures and Tables

**Table 1:** Primer sequences used for RT-qPCR.

Gene Name	Primer Direction	Primer Sequence (5′ to 3′)
*β-actin*	Forward	TTGGCAATGAGCGGTT
	Reverse	AGTTGAAGGTAGTTTCGTGGAT
*IL-1β*	Forward	GCACGATGCACCTGTACGAT
	Reverse	TGGAGAACACCACTTGTTGC
*IL-10*	Forward	TCAAGGCGCATGTGAACTCC
	Reverse	GATGTCAAACTCACTCATGGCT
*TNF*	Forward	GCTGCACTTTGGAGTGATCG
	Reverse	GGGTTTGCTACAACATGGGC
*TGF-β2*	Forward	CAGCACACTCGATATGGACCA
	Reverse	CCTCGGGCTCAGGATAGTCT
*IL-6*	forward	AATCATCACTGGTCTTTTGGAG
	reverse	GCATTTGTGGTTGGGTCA
*IL-8*	forward	GACATACTCCAAACCTTTCCACC
	reverse	AACTTCTCCACAACCCTCTGC
*CXCL5*	forward	AGCTGCGTTGCGTTTGTTTAC
	reverse	TGGCGAACACTTGCAGATTAC
*FOXO1*	Forward	TTATGACCGAACAGGATGATCTTG
	Reverse	TGTTGGTGATGAGAGAAGGTTGAG
*BCL2L11*	Forward	TAAGTTCTGAGTGTGACCGAGA
	Reverse	GCTCTGTCTGTAGGGAGGTAGG
*BCL6*	Forward	GGAGTCGAGACATCTTGACTGA
	Reverse	ATGAGGACCGTTTTATGGGCT
*S1PR1*	Forward	GCCTCTTCCTGCTAATCAGCG
	Reverse	GCAGTACAGAATGACGATGGAG
*TRAIL*	Forward	TGCGTGCTGATCGTGATCTTC
	Reverse	GCTCGTTGGTAAAGTACACGTA
*KLF2*	Forward	TTCGGTCTCTTCGACGACG
	Reverse	TGCGAACTCTTGGTGTAGGTC

## Data Availability

The RNA-seq datasets generated in this study have been deposited in NCBI’s Gene Expression Omnibus (https://www.ncbi.nlm.nih.gov/geo/query/acc.cgi, accessed on 17 May 2022) for access through the GEO series accession number GSE184777.

## Supplementary Materials

The Supplementary materials are available online at https://www.mdpi.com/article/10.3390/ijms23105728/s1.
