# Supplementary material for: RNA Sequencing Reveals the Upregulation of FOXO Signaling Pathway in Porphyromonas gingivalis Persister-Treated Human Gingival Epithelial Cells
Source: Int J Mol Sci. 2022 May 20;23(10):5728. doi: 10.3390/ijms23105728 (PMC9146424; doi:10.3390/ijms23105728)
Supplement: Supplementary file 1 [file ijms-23-05728-s001.zip › Supplementary files/Supporting figures.pdf]

RNA Sequencing Reveals the Upregulation of FOXO Signaling Pathway in *Porphyromonas gingivalis* Persister-Treated Human Gingival Epithelial Cells

Chuan Wang, Xuan Li, Tianfan Cheng, Leilei Wang and Lijian Jin[#]

Faculty of Dentistry, The University of Hong Kong, Hong Kong SAR, China

Correspondence

[#]Prof. Lijian Jin, Division of Periodontology & Implant Dentistry, Faculty of Dentistry, The University of Hong Kong, 34 Hospital Road, Hong Kong SAR, China.

Email: ljjin@hku.hk

Data availability

Data that support the findings of this study can be found in the Supporting Information of this article. The RNA-seq datasets generated in this study have been deposited in NCBI's Gene Expression Omnibus (https://www.ncbi.nlm.nih.gov/geo/query/acc.cgi, accessed on 19 May 2022) for access through the GEO series accession number (GSE184777).

Funding information

This study was supported by the Hong Kong Research Grants Council (GRF No. 17122918 and 17119819) and the Modern Dental Laboratory/HKU Endowment Fund to L.J. Jin.

Conflict of interest

The authors declare that they have no conflict of interest in connection with this article.

Supporting figures

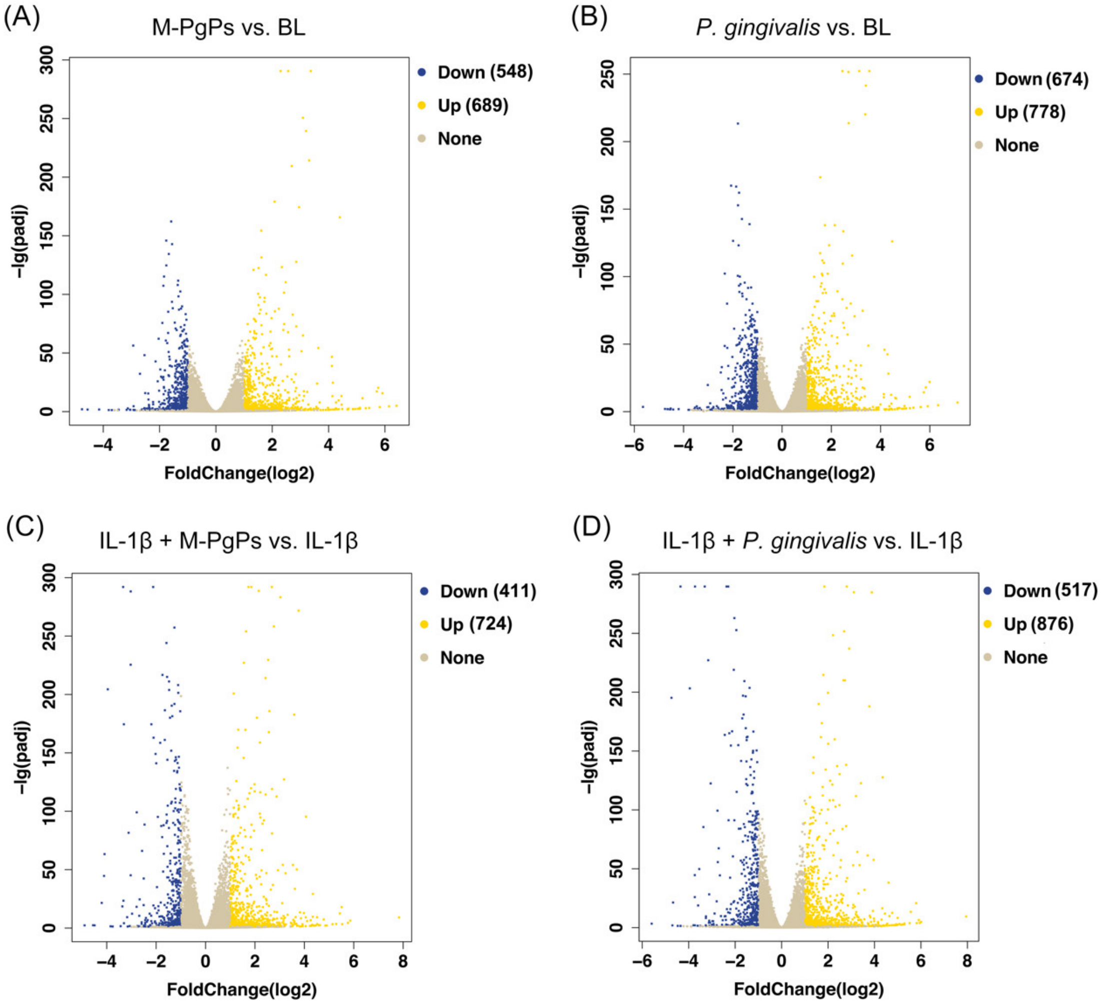

Figure S1 Volcano plot of global DEGs in different comparison groups. M-PgPs vs. BL (A), *P. gingivalis* vs. BL (B), IL-1β + M-PgPs vs. IL-1β (C), and IL-1β + *P. gingivalis* vs. IL-1β (D). Blue dots represent significantly down-regulated genes ($P < 0.05$, Log2 fold change ≤-1). Yellow dots represent significantly up-regulated genes ($P < 0.05$, Log2 fold change ≥1). Brown dots represent insignificantly differential expressed genes. Samples for RNA-Seq were obtained from three independent experiments.

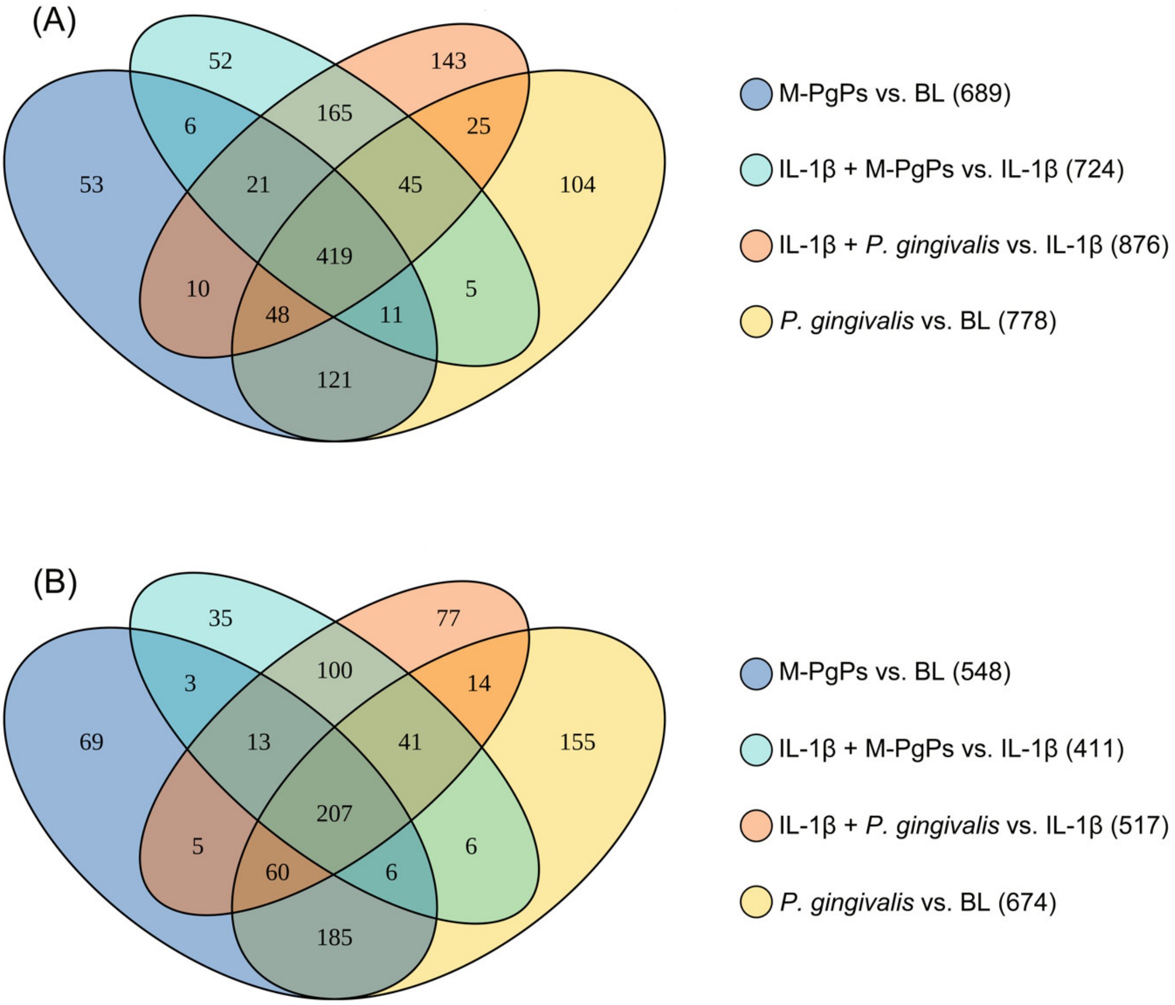

Figure S2 Venn diagram of global DEGs in different comparison groups. (A) Up-regulated genes. (B) Down-regulated genes. Samples for RNA-Seq were obtained from three independent experiments.

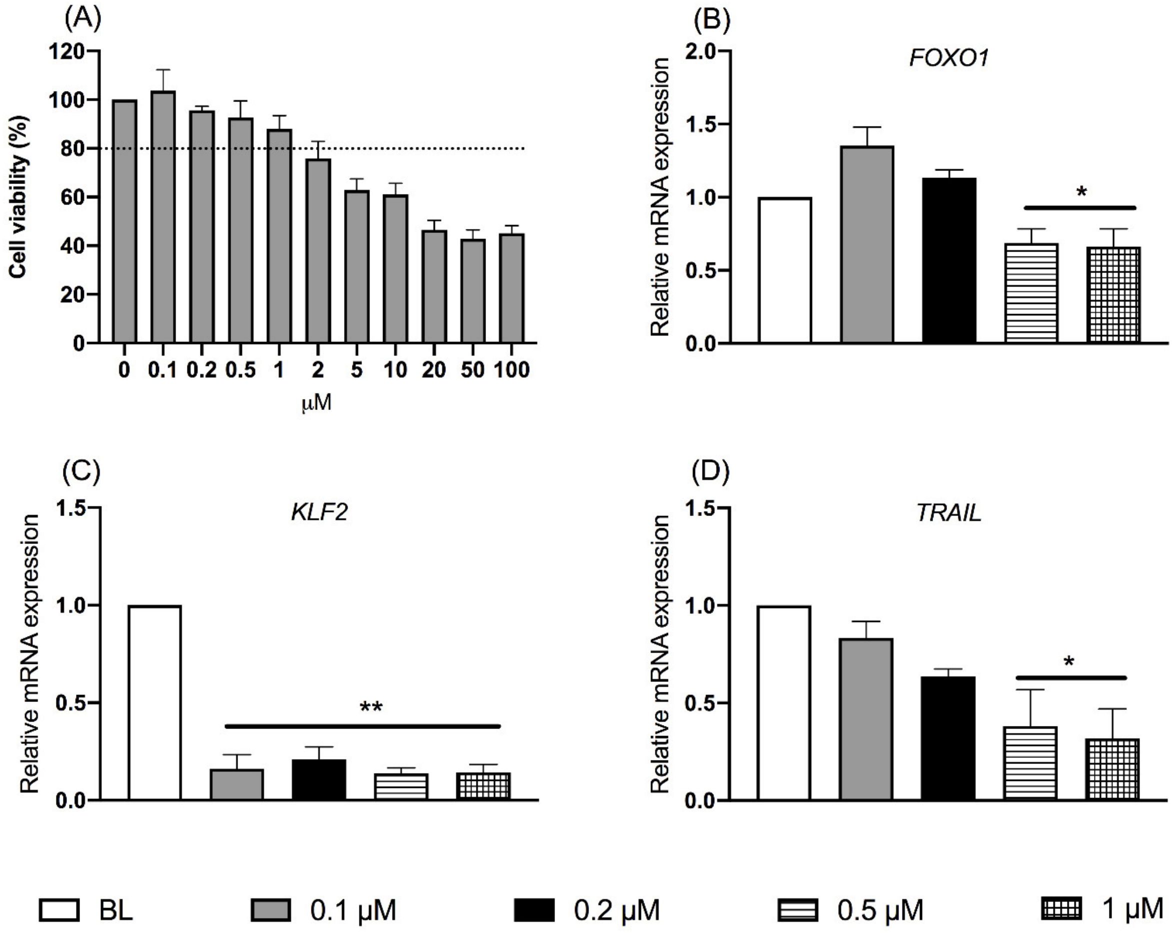

Figure S3 FOXO1 inhibitor (Fi) concentration test. HGECs were treated with different concentrations of Fi (0 μM to 100 μM) for 24 h. (A) Cell viability tested by MTT assay. (B) to (D) Relative mRNA expression levels of *FOXO1*, *KLF2* and *TRAIL* were determined by qPCR. * $P < 0.01$; ** $P < 0.001$ compared with BL. Data represent the mean ± SD of three independent experiments.

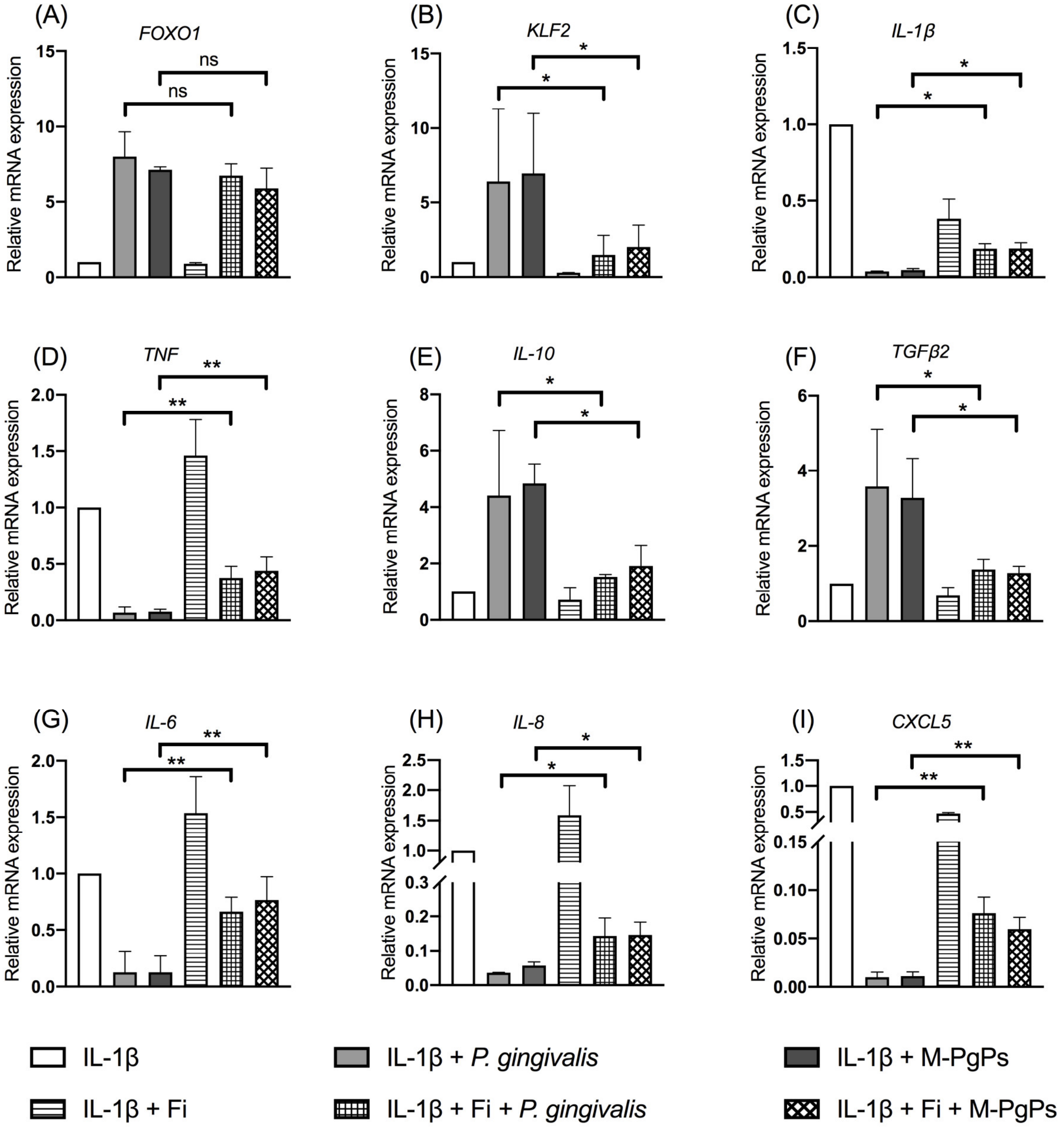

Figure S4 FOXO1 inhibitor (Fi) rescues the inhibitory effects of M-PgPs on the immuno-inflammatory response of HGECs under inflammatory condition. Relative mRNA expression levels of *FOXO1* (A), *KLF2* (B), *IL-1β* (C), *TNF* (D), *IL-10* (E), *TGF-β2* (F), IL-6 (G), *IL-8* (H) and *CXCL5* (I) in HGECs after 24-h treatment of M-PgPs (1:100) were analyzed by qPCR. HGECs treated with *P. gingivalis* (1:100, 24 h) served as the positive control. IL-1β (1 ng/ml) was added 6 h prior to the treatments of *P. gingivalis* and M-PgPs, while Fi (1 μM) was added 2 h before M-PgPs and *P. gingivalis* treatments. ns, no significance; * $P < 0.05$; ** $P < 0.01$. Data represent the mean ± SD of three independent experiments.
