# Supplementary material for: RNA Sequencing Reveals the Upregulation of FOXO Signaling Pathway in Porphyromonas gingivalis Persister-Treated Human Gingival Epithelial Cells
Source: Int J Mol Sci. 2022 May 20;23(10):5728. doi: 10.3390/ijms23105728 (PMC9146424; doi:10.3390/ijms23105728)
Supplement: Supplementary file 1 [file ijms-23-05728-s001.zip › Supplementary files/Table S1.pdf]

Table S1 The top 20 DEGs similarly identified in the four comparison groups

**The top 20 upregulated genes (based on the Log2 fold change of M-PgPs vs BL)**					
Gene ID	Gene Name	Log2 Fold Change			
		M-PgPs vs. BL	*P. gingivalis* vs. BL	IL-1β + M-PgPs vs. IL-1β	IL-1β + *P. gingivalis* vs. IL-1β
ENSG00000165272	AQP3	4.3989324	4.4712577	4.0658647	4.3527156
ENSG00000156804	FBXO32	3.3677506	3.5436826	2.7649915	2.9119453
ENSG00000163347	CLDN1	3.3113900	3.3812388	2.5847055	2.7225392
ENSG00000171346	KRT15	3.2009895	3.2000906	3.7679138	3.8835062
ENSG00000184113	CLDN5	3.0890181	3.1340865	3.0321418	3.1106725
ENSG00000148926	ADM	2.9501664	3.4027730	1.8394358	2.0073592
ENSG00000170271	FAXDC2	2.8522659	2.8428846	3.5889795	3.7786953
ENSG00000180573	HIST1H2AC	2.6889626	2.7042439	2.5626884	2.6647273
ENSG00000185909	KLHDC8B	2.5671331	2.6950338	2.5366656	2.6896529

ENSG00000164120	HPGD	2.4769272	2.4654177	2.6926484	2.7796053
ENSG00000102760	RGCC	2.4182401	2.4827123	2.8747440	3.2042561
ENSG00000141738	GRB7	2.3403069	2.4879869	2.2037920	2.2856637
ENSG00000157514	TSC22D3	2.2969590	2.4523933	2.6846517	2.8050771
ENSG00000100100	PIK3IP1	2.0832630	1.5522600	1.4287081	1.4642139
ENSG00000170412	GPRC5C	1.7768729	1.9121728	1.5789644	1.6480662
ENSG00000107262	BAG1	1.6184454	1.7396585	1.6144949	3.7786953
ENSG00000135821	GLUL	1.6099119	1.5463754	1.7375424	1.8410556
ENSG00000176046	NUPR1	1.5165685	1.6973238	1.8522223	1.9969648
ENSG00000151632	AKR1C2	1.5006107	1.5522600	1.3231291	1.3745780
ENSG00000189221	MAOA	1.3363730	1.3044064	1.1425614	1.2189908

Top 20 down-regulated genes (based on the Log2 fold change of M-PgPs vs BL)

Gene ID	Gene Name	Log2 Fold Change			

		M-PgPs vs. BL	*P. gingivalis* vs. BL	IL-1β + M-PgPs vs. IL-1β	IL-1β + *P. gingivalis* vs. IL-1β
ENSG00000 171617	ENC1	-2.3268292	-1.8995164	-1.7545489	-1.9341888
ENSG00000 112972	HMGCS1	-2.0692747	-1.7628486	-1.6888997	-1.9477261
ENSG00000 172115	CYCS	-1.9893248	-1.8407531	-1.8358900	-2.0595387
ENSG00000 087494	PTHLH	-1.8638933	-1.6718268	-1.5432711	-1.6349458
ENSG00000 110092	CCND1	-1.7920013	-1.5869896	-1.2159890	-1.3516549
ENSG00000 197451	HNRNPAB	-1.7888619	-1.5550501	-1.3888501	-1.5050150
ENSG00000 115008	IL1A	-1.7649250	-1.7600241	-2.1189747	-2.3718589
ENSG00000 187678	SPRY4	-1.7486534	-1.5309880	-1.3665313	-1.5216728
ENSG00000 168906	MAT2A	-1.7450236	-1.3551784	-1.0564000	-1.1385422
ENSG00000 181649	PHLDA2	-1.7322295	-1.6920877	-1.3914789	-1.4721959
ENSG00000 101670	LIPG	-1.7147466	-1.5594333	-1.6504637	-1.8641808

ENSG00000 117525	F3	-1.6186853	-1.4603853	-1.6458314	-1.7419959
ENSG00000 152952	PLOD2	-1.5182557	-1.3388854	-1.3695957	-1.5010581
ENSG00000 175592	FOSL1	-1.4753451	-1.4303079	-1.2413161	-1.4041137
ENSG00000 131473	ACLY	-1.4439539	-1.2375327	-1.1421869	-1.2364041
ENSG00000 177283	FZD8	-1.3924709	-1.6889459	-1.1354117	-1.3666048
ENSG00000 163235	TGFA	-1.3474170	-1.2457886	-1.4733297	-1.6932265
ENSG00000 123505	AMD1	-1.3238774	-1.1323272	-1.0846984	-1.2378600
ENSG00000 134954	ETS1	-1.2772555	-1.1156367	-1.2588583	-1.3790420
ENSG00000 149428	HYOU1	-1.0497075	-1.0472391	-1.0437086	-1.1809964
