# Supplementary material for: RNA Sequencing Reveals the Upregulation of FOXO Signaling Pathway in Porphyromonas gingivalis Persister-Treated Human Gingival Epithelial Cells
Source: Int J Mol Sci. 2022 May 20;23(10):5728. doi: 10.3390/ijms23105728 (PMC9146424; doi:10.3390/ijms23105728)
Supplement: Supplementary file 1 [file ijms-23-05728-s001.zip › Supplementary files/Table S2.pdf]

Table S2 Numbers of terms significantly enriched within each category.

	M-PgPs vs. BL	*P. gingivalis* vs. BL	IL-1β + M-PgPs Vs. IL-1β	IL-1β + *P. gingivalis* Vs. IL-1β
CC	24	31	55	71
BP	712	996	1238	1310
MF	54	77	95	120
