# Supplementary material for: RNA Sequencing Reveals the Upregulation of FOXO Signaling Pathway in Porphyromonas gingivalis Persister-Treated Human Gingival Epithelial Cells
Source: Int J Mol Sci. 2022 May 20;23(10):5728. doi: 10.3390/ijms23105728 (PMC9146424; doi:10.3390/ijms23105728)
Supplement: Supplementary file 1 [file ijms-23-05728-s001.zip › Supplementary files/Table S3.pdf]

Table S3 The top 20 enriched GO terms in the four comparison groups.

The top 20 enriched GO terms				
**M-PgPs vs. BL**				
**Cellular Component**				
Term ID	Term name	p-value	FDR	Rich_Ratio
GO:0016020	membrane	0.001544772	0.048724673	1.117390787
GO:0044425	membrane part	0.000462095	0.017490313	1.150084694
GO:0016021	integral component of membrane	0.000108129	0.005456906	1.206886141
GO:0031224	intrinsic component of membrane	6.51E-05	0.00391068	1.210726359
GO:0044421	extracellular region part	7.09E-05	0.00391068	1.288610629
GO:0005886	plasma membrane	1.66E-06	0.000208995	1.298291045
GO:0098588	bounding membrane of organelle	0.000723392	0.024891271	1.350804467
GO:0005576	extracellular region	2.44E-06	0.00026351	1.526734751
GO:0005794	Golgi apparatus	0.000635668	0.022914304	1.56619592
GO:0044459	plasma membrane part	9.53E-15	7.22E-12	1.734717482
GO:0005615	extracellular space	4.89E-08	7.41E-06	1.745472876
GO:0009986	cell surface	0.000337542	0.013569589	1.760158035
GO:0005911	cell-cell junction	0.000340584	0.013569589	1.802976378
GO:0098590	plasma membrane region	4.01E-09	7.59E-07	1.961629174
GO:0005887	integral component of plasma membrane	1.79E-12	4.52E-10	2.033848701

GO:0031226	intrinsic component of plasma membrane	2.21E-14	8.35E-12	2.092117665
GO:0005788	endoplasmic reticulum lumen	0.000842157	0.027717953	2.123444396
GO:0016324	apical plasma membrane	0.000221253	0.01002513	2.129476909
GO:0043235	receptor complex	5.07E-05	0.003837522	2.328946897
GO:0044815	DNA packaging complex	7.23E-05	0.00391068	2.852508603

**Biological Progress**				
Term ID	Term name	p-value	FDR	Rich_Ratio
GO:0060255	regulation of macromolecule metabolic process	0.002997535	0.033469096	1.139183582
GO:0051171	regulation of nitrogen compound metabolic process	0.000407828	0.007999238	1.17834463
GO:0019222	regulation of metabolic process	3.90E-05	0.001210112	1.189589208
GO:2000112	regulation of cellular macromolecule biosynthetic process	0.001858006	0.023940384	1.196057866
GO:0031326	regulation of cellular biosynthetic process	0.001152953	0.017125893	1.1991352
GO:0051252	regulation of RNA metabolic process	0.002532461	0.029471066	1.199256508

GO:0010556	regulation of macromolecule biosynthetic process	0.001354817	0.018973174	1.200369753
GO:0019219	regulation of nucleobase-containing compound metabolic process	0.000887897	0.014027408	1.209332565
GO:0080090	regulation of primary metabolic process	2.14E-05	0.000725174	1.21464257
GO:0009889	regulation of biosynthetic process	0.000261592	0.005725564	1.224667024
GO:0031323	regulation of cellular metabolic process	7.08E-06	0.000314162	1.226607476
GO:0065007	biological regulation	6.57E-16	7.24E-13	1.230590315
GO:0050789	regulation of biological process	8.93E-15	7.38E-12	1.233448056
GO:0051246	regulation of protein metabolic process	0.001833197	0.023940384	1.249609477
GO:0043412	macromolecule modification	0.001389317	0.019330307	1.249644425
GO:0050794	regulation of cellular process	1.11E-14	8.15E-12	1.252693975
GO:2001141	regulation of RNA biosynthetic process	0.000262931	0.00573113	1.257781596
GO:0036211	protein modification process	0.001445032	0.019899294	1.258054091

GO:0006464	cellular protein modification process	0.001445032	0.019899294	1.258054091
GO:1903506	regulation of nucleic acid-templated transcription	0.000222316	0.005063624	1.26168205

Molecular Function

Term ID	Term name	p-value	FDR	Rich_Ratio
GO:0005515	protein binding	1.69E-07	0.000243742	1.228411104
GO:0097367	carbohydrate derivative binding	0.000761161	0.027559296	1.290193119
GO:0042802	identical protein binding	0.00084454	0.027559296	1.321928093
GO:0003700	DNA binding transcription factor activity	0.000620007	0.024146767	1.363365539
GO:0004871	signal transducer activity	0.000600382	0.024146767	1.389246879
GO:0016772	transferase activity, transferring phosphorus-containing groups	0.000612961	0.024146767	1.441627298
GO:0005509	calcium ion binding	0.001859023	0.049608381	1.573589362
GO:0005102	receptor binding	2.58E-06	0.001239321	1.578782527
GO:0003690	double-stranded DNA binding	0.000294181	0.014617775	1.609246393
GO:0032403	protein complex binding	0.00169445	0.04606986	1.642006291
GO:0043565	sequence-specific DNA binding	9.22E-06	0.001568905	1.674931385
GO:0016301	kinase activity	1.00E-05	0.001568905	1.737290142

GO:0001067	regulatory region nucleic acid binding	1.94E-05	0.001749836	1.742938196
GO:0044212	transcription regulatory region DNA binding	1.81E-05	0.001749836	1.747263104
GO:0016773	phosphotransferase activity, alcohol group as acceptor	1.20E-05	0.001568905	1.780950696
GO:0004672	protein kinase activity	3.10E-05	0.002565334	1.817153629
GO:1990837	sequence-specific double-stranded DNA binding	1.56E-05	0.001726497	1.828233834
GO:0000976	transcription regulatory region sequence-specific DNA binding	1.07E-05	0.001568905	1.890848226
GO:0000977	RNA polymerase II regulatory region sequence-specific DNA binding	3.38E-05	0.002565334	1.906994246
GO:0001012	RNA polymerase II regulatory region DNA binding	3.38E-05	0.002565334	1.906994246

***P. gingivalis* vs. BL**

Cellular Component

Term ID	Term name	p-value	FDR	Rich_Ratio
GO:0044421	extracellular region part	0.000850752	0.032096554	1.218201922
GO:0005886	plasma membrane	2.90E-05	0.002674858	1.236691361

GO:0098588	bounding membrane of organelle	0.000732987	0.030122968	1.321991043
GO:0044463	cell projection part	0.001721469	0.047627306	1.347465614
GO:0120038	plasma membrane bounded cell projection part	0.001721469	0.047627306	1.347465614
GO:0030054	cell junction	0.000969408	0.034982977	1.372694207
GO:0044433	cytoplasmic vesicle part	0.000668807	0.029396294	1.388694528
GO:0005576	extracellular region	6.22E-07	7.38E-05	1.514402169
GO:0005794	Golgi apparatus	0.000184865	0.013948878	1.57648598
GO:0005622	intracellular	0.001620784	0.047627306	1.593390771
GO:0005911	cell-cell junction	0.001828168	0.048947733	1.623130222
GO:0005615	extracellular space	4.00E-07	5.54E-05	1.629234807
GO:0044459	plasma membrane part	1.50E-14	1.25E-11	1.668331447
GO:0031253	cell projection membrane	0.000451138	0.024962952	1.880533066
GO:0005887	integral component of plasma membrane	2.98E-11	8.24E-09	1.885463934
GO:0098590	plasma membrane region	4.40E-10	9.14E-08	1.93978429
GO:0016324	apical plasma membrane	0.000511836	0.026551509	1.958624787
GO:0031226	intrinsic component of plasma membrane	8.86E-14	3.68E-11	1.972216184
GO:0009986	cell surface	3.49E-07	5.54E-05	2.04560278
GO:0005788	endoplasmic reticulum lumen	0.000562482	0.027462374	2.066342454
**Biological Progress**				

Term ID	Term name	p-value	FDR	Rich_Ratio
GO:0010468	regulation of gene expression	0.000256637	0.004251922	1.193472438
GO:0060255	regulation of macromolecule metabolic process	6.63E-06	0.000193295	1.203837674
GO:0065007	biological regulation	3.65E-20	4.45E-17	1.242584706
GO:0019222	regulation of metabolic process	1.37E-08	9.10E-07	1.246588272
GO:0050789	regulation of biological process	3.00E-19	2.44E-16	1.24961988
GO:0050896	response to stimulus	2.80E-07	1.18E-05	1.256142113
GO:0051171	regulation of nitrogen compound metabolic process	9.12E-08	4.58E-06	1.25734436
GO:0050794	regulation of cellular process	3.11E-20	4.45E-17	1.27881698
GO:2000112	regulation of cellular macromolecule biosynthetic process	2.75E-06	9.14E-05	1.284856336
GO:0010556	regulation of macromolecule biosynthetic process	1.65E-06	5.61E-05	1.288265971
GO:0051252	regulation of RNA metabolic process	3.92E-06	0.000125388	1.294870459

GO:0019219	regulation of nucleobase-containing compound metabolic process	8.94E-07	3.24E-05	1.296744687
GO:0080090	regulation of primary metabolic process	4.47E-10	3.99E-08	1.297848651
GO:0031323	regulation of cellular metabolic process	2.77E-10	2.60E-08	1.299893454
GO:0002376	immune system process	0.000377456	0.005769929	1.3019376
GO:0031326	regulation of cellular biosynthetic process	2.69E-07	1.15E-05	1.304158508
GO:0051246	regulation of protein metabolic process	5.62E-05	0.00121024	1.30712948
GO:0080134	regulation of response to stress	0.003909866	0.033724324	1.308196915
GO:0043412	macromolecule modification	3.25E-05	0.000754645	1.308634855
GO:0036211	protein modification process	5.32E-05	0.001153838	1.310620953

Molecular Function

Term ID	Term name	p-value	FDR	Rich_Ratio
GO:0005488	binding	0.000654424	0.021146072	1.062499191
GO:0043169	cation binding	0.001348602	0.029881169	1.160418597
GO:0043167	ion binding	4.49E-05	0.002900776	1.163708958
GO:0046872	metal ion binding	0.000781643	0.023313994	1.170887531
GO:0005515	protein binding	5.38E-08	2.78E-05	1.219342011

GO:0043168	anion binding	0.001003861	0.027172172	1.23082706
GO:0016740	transferase activity	0.000957675	0.027006441	1.250033323
GO:0003677	DNA binding	0.000195455	0.008661429	1.261019131
GO:0097367	carbohydrate derivative binding	0.000134312	0.007183391	1.307768019
GO:0042802	identical protein binding	7.20E-05	0.00429375	1.360242979
GO:0098772	molecular function regulator	0.00015861	0.007806899	1.374034105
GO:0016772	transferase activity, transferring phosphorus-containing groups	0.001050484	0.027172172	1.385067854
GO:0140110	transcription regulator activity	8.06E-06	0.000735564	1.400624324
GO:0000981	RNA polymerase II transcription factor activity, sequence-specific DNA binding	4.40E-05	0.002900776	1.432205846
GO:0005102	receptor binding	6.50E-05	0.004031137	1.442099122
GO:0003700	DNA binding transcription factor activity	3.64E-07	6.48E-05	1.520961554
GO:0008134	transcription factor binding	0.000827609	0.023770761	1.562533154
GO:0016301	kinase activity	0.000119867	0.006639793	1.576521794

GO:0016773	phosphotransferase activity, alcohol group as acceptor	0.000170976	0.00803588	1.600096271
GO:0004672	protein kinase activity	0.000425082	0.015255637	1.615943379

IL-1β + M-PgPs vs. IL-1β

Cellular Component

Term ID	Term name	p-value	FDR	Rich_Ratio
GO:0044425	membrane part	0.000407641	0.009756212	1.157619671
GO:0005737	cytoplasm	0.003144943	0.042605079	1.167383583
GO:0016021	integral component of membrane	0.000286061	0.008441149	1.200107562
GO:0031224	intrinsic component of membrane	8.73E-05	0.003686305	1.214848371
GO:0005886	plasma membrane	1.91E-07	1.52E-05	1.339489256
GO:0031982	vesicle	8.57E-06	0.000473274	1.343206947
GO:0098588	bounding membrane of organelle	0.000238539	0.007785044	1.401640668
GO:0097708	intracellular vesicle	0.000375988	0.009308951	1.468707303
GO:0031410	cytoplasmic vesicle	0.000354198	0.009082654	1.471618517
GO:0030054	cell junction	0.000149226	0.005357196	1.496101445
GO:0044421	extracellular region part	6.87E-12	8.22E-10	1.544512142
GO:0005856	cytoskeleton	0.000572235	0.011188316	1.565884086
GO:0005794	Golgi apparatus	0.000562425	0.011188316	1.596626971
GO:0044433	cytoplasmic vesicle part	2.50E-06	0.000163452	1.638184707
GO:0012506	vesicle membrane	0.000956322	0.015968356	1.670127996

GO:0005911	cell-cell junction	0.002283548	0.032791752	1.691386329
GO:0030659	cytoplasmic vesicle membrane	0.000523723	0.011188316	1.722436255
GO:0044459	plasma membrane part	8.42E-17	2.53E-14	1.82837797
GO:0005622	intracellular	9.58E-05	0.003822116	1.868759724
GO:0098589	membrane region	0.001256865	0.020509757	1.900016741

Biological Progress

Term ID	Term name	p-value	FDR	Rich Ratio
GO:0009987	cellular process	7.69E-05	0.001051974	1.066690297
GO:0010468	regulation of gene expression	0.000535409	0.005372173	1.205553269
GO:0060255	regulation of macromolecule metabolic process	1.86E-06	4.33E-05	1.244465196
GO:0019222	regulation of metabolic process	7.45E-08	2.64E-06	1.262823516
GO:0044281	small molecule metabolic process	0.0079846	0.045465643	1.265946516
GO:0006796	phosphate-containing compound metabolic process	0.00822178	0.046467675	1.27513042
GO:0043412	macromolecule modification	0.000641859	0.006229865	1.279893767
GO:0065007	biological regulation	1.57E-22	1.07E-19	1.289194305

GO:0050789	regulation of biological process	1.59E-20	5.73E-18	1.290514996
GO:2000112	regulation of cellular macromolecule biosynthetic process	1.72E-05	0.000298374	1.292781594
GO:0019219	regulation of nucleobase-containing compound metabolic process	1.21E-05	0.000220505	1.295662422
GO:0010556	regulation of macromolecule biosynthetic process	1.05E-05	0.000193501	1.297316274
GO:0031326	regulation of cellular biosynthetic process	5.27E-06	0.000108389	1.301236316
GO:0051171	regulation of nitrogen compound metabolic process	7.40E-09	3.11E-07	1.315974372
GO:0009889	regulation of biosynthetic process	1.36E-06	3.26E-05	1.317821209
GO:0036211	protein modification process	0.000145974	0.001813534	1.327880067
GO:0006464	cellular protein modification process	0.000145974	0.001813534	1.327880067
GO:0051252	regulation of RNA metabolic process	5.47E-06	0.000111311	1.327882999

GO:0050794	regulation of cellular process	5.61E-22	2.78E-19	1.32805914
GO:0031323	regulation of cellular metabolic process	4.00E-10	2.06E-08	1.335392068

Molecular Function

Term ID	Term name	p-value	FDR	Rich_Ratio
GO:0005488	binding	0.000146458	0.004840103	1.078952436
GO:0097367	carbohydrate derivative binding	0.001006845	0.020867243	1.293887219
GO:0140110	transcription regulator activity	0.001477455	0.029295825	1.309010211
GO:0019899	enzyme binding	0.000296672	0.007918864	1.335352677
GO:0000981	RNA polymerase II transcription factor activity, sequence-specific DNA binding	0.00210959	0.036173138	1.353492506
GO:0005515	protein binding	4.49E-15	2.08E-12	1.362667305
GO:0042802	identical protein binding	0.000217416	0.006858471	1.376593968
GO:0003700	DNA binding transcription factor activity	0.000142427	0.004827167	1.426691746
GO:0004872	receptor activity	0.000258221	0.007314517	1.435701637
GO:0004888	transmembrane signaling receptor activity	0.000705509	0.016118256	1.445309179
GO:0099600	transmembrane receptor activity	0.000705509	0.016118256	1.445309179

GO:0046983	protein dimerization activity	0.000116513	0.004479247	1.449311893
GO:0060089	molecular transducer activity	0.000104473	0.004143104	1.460042363
GO:0038023	signaling receptor activity	2.97E-05	0.00147466	1.524961546
GO:0004871	signal transducer activity	5.93E-06	0.000357838	1.555320617
GO:0008092	cytoskeletal protein binding	0.000190215	0.006139949	1.586626891
GO:0016301	kinase activity	0.000254457	0.007314517	1.61831856
GO:0016773	phosphotransferase activity, alcohol group as acceptor	0.00023714	0.007155447	1.665084968
GO:0098772	molecular function regulator	2.58E-10	5.98E-08	1.753578801
GO:0004672	protein kinase activity	3.69E-05	0.001697726	1.842565143

IL-1β + *P. gingivalis* vs. IL-1β

Cellular Component

Term ID	Term name	p-value	FDR	Rich_Ratio
GO:0016020	membrane	3.17E-05	0.0009779	1.14876665
GO:0005737	cytoplasm	0.000238827	0.005377016	1.193306994
GO:0044425	membrane part	1.93E-06	8.94E-05	1.196537891
GO:0016021	integral component of membrane	7.99E-06	0.000331525	1.226589144
GO:0031224	intrinsic component of membrane	1.26E-06	6.21E-05	1.243623762

GO:0043230	extracellular organelle	0.001006016	0.014680378	1.275710493
GO:1903561	extracellular vesicle	0.000972214	0.014454809	1.276769759
GO:0070062	extracellular exosome	0.000799315	0.013401286	1.282805657
GO:0031982	vesicle	1.24E-06	6.21E-05	1.339242459
GO:0120038	plasma membrane bounded cell projection part	0.001906911	0.024236219	1.349793537
GO:0044463	cell projection part	0.001906911	0.024236219	1.349793537
GO:0005886	plasma membrane	1.35E-09	1.33E-07	1.359703087
GO:0098805	whole membrane	0.00085404	0.013734355	1.400160609
GO:0098588	bounding membrane of organelle	1.76E-05	0.000660698	1.430129828
GO:0097458	neuron part	0.000133798	0.003194929	1.434419183
GO:0097708	intracellular vesicle	0.000116286	0.002863541	1.461282028
GO:0031410	cytoplasmic vesicle	0.000108275	0.002752279	1.464178523
GO:0044421	extracellular region part	1.50E-13	2.37E-11	1.529804138
GO:0030054	cell junction	5.06E-06	0.000221465	1.548758227
GO:0005794	Golgi apparatus	0.000360637	0.007104549	1.556135489

**Biological Progress**				
Term ID	Term name	p-value	FDR	Rich_Ratio
GO:0009987	cellular process	0.000306417	0.003446248	1.054840607
GO:0010468	regulation of gene expression	0.000144803	0.001818217	1.205529137

GO:0060255	regulation of macromolecule metabolic process	2.20E-07	6.81E-06	1.240935027
GO:0044281	small molecule metabolic process	0.005406151	0.03462835	1.253307245
GO:0031326	regulation of cellular biosynthetic process	1.36E-05	0.000254173	1.258120754
GO:0019222	regulation of metabolic process	4.41E-09	1.98E-07	1.259801952
GO:2000112	regulation of cellular macromolecule biosynthetic process	1.98E-05	0.000348488	1.26157082
GO:0006796	phosphate-containing compound metabolic process	0.005174404	0.033430756	1.264813423
GO:0050789	regulation of biological process	5.42E-21	1.81E-18	1.266120542
GO:0010556	regulation of macromolecule biosynthetic process	1.16E-05	0.000222067	1.266305254
GO:0019219	regulation of nucleobase-containing compound metabolic process	9.19E-06	0.000179797	1.270213604
GO:0065007	biological regulation	2.48E-24	1.82E-21	1.272845377

GO:0009889	regulation of biosynthetic process	2.66E-06	5.97E-05	1.277547674
GO:0051171	regulation of nitrogen compound metabolic process	1.33E-08	5.52E-07	1.279554731
GO:0031323	regulation of cellular metabolic process	2.81E-10	1.62E-08	1.305062425
GO:0050794	regulation of cellular process	3.82E-23	2.23E-20	1.305097784
GO:0043412	macromolecule modification	5.04E-05	0.000760879	1.305661626
GO:0080090	regulation of primary metabolic process	6.14E-11	4.09E-09	1.318686966
GO:0036211	protein modification process	4.66E-05	0.000707986	1.318890703
GO:0006464	cellular protein modification process	4.66E-05	0.000707986	1.318890703

Molecular Function

Term ID	Term name	p-value	FDR	Rich_Ratio
GO:0005488	binding	2.74E-05	0.001507438	1.07935713
GO:0043167	ion binding	0.001489175	0.026377234	1.126222163
GO:0043168	anion binding	0.000132944	0.00539121	1.278248291
GO:0003700	DNA binding transcription factor activity	0.002456002	0.036744649	1.29499436
GO:0019899	enzyme binding	0.000227806	0.007313534	1.308066488

GO:0097367	carbohydrate derivative binding	0.000138688	0.005479954	1.312563406
GO:0004872	receptor activity	0.00230739	0.034859683	1.317359135
GO:0060089	molecular transducer activity	0.001270022	0.023298859	1.334076332
GO:0005515	protein binding	1.46E-16	2.26E-13	1.345226957
GO:0046983	protein dimerization activity	0.000539481	0.01385567	1.357050641
GO:0038023	signaling receptor activity	0.000458833	0.012404586	1.386060918
GO:0042802	identical protein binding	1.40E-05	0.000937138	1.405622228
GO:0030234	enzyme regulator activity	0.001566421	0.02670623	1.40905318
GO:0004871	signal transducer activity	3.21E-05	0.001703407	1.452868467
GO:0016301	kinase activity	0.001538416	0.02670623	1.468499244
GO:0008289	lipid binding	0.003329061	0.044609421	1.483260648
GO:0005509	calcium ion binding	0.001618995	0.026826565	1.543831707
GO:0016773	phosphotransferase activity, alcohol group as acceptor	0.000598128	0.014630411	1.550134967
GO:0008092	cytoskeletal protein binding	1.39E-05	0.000937138	1.625938837
GO:0004857	enzyme inhibitor activity	0.003143996	0.043590876	1.629736492
