# Supplementary material for: RNA Sequencing Reveals the Upregulation of FOXO Signaling Pathway in Porphyromonas gingivalis Persister-Treated Human Gingival Epithelial Cells
Source: Int J Mol Sci. 2022 May 20;23(10):5728. doi: 10.3390/ijms23105728 (PMC9146424; doi:10.3390/ijms23105728)
Supplement: Supplementary file 1 [file ijms-23-05728-s001.zip › Supplementary files/Table S4.pdf]

Table S4 Numbers of genes mapped into the signaling pathways significantly enriched in the four comparison groups.

		M-PgPs vs. BL		*P. gingivalis* vs. BL		IL-1β + M-PgPs vs. IL-1β		IL-1β + *P. gingivalis* vs. IL-1β	
Name	Map	Up	Down	Up	Down	Up	Down	Up	Down
Cytokine-cytokine receptor interaction	map04060	12	15	12	20	14	22	17	22
Hematopoietic cell lineage	map04640	6	9	7	8	6	10	7	12
MAPK signaling pathway	map04010	18	13	18	18	14	11	18	13
MicroRNAs in cancer	map05206	9	9	9	10	12	7	12	8
Rap1 signaling pathway	map04015	17	7	16	9	12	7	17	7
Rheumatoid arthritis	map05323	7	7	7	9	4	14	8	14
TNF signaling pathway	map04668	2	13	2	16	2	19	2	19
IL-17 signaling pathway	map04657	0	14	0	16	1	22	1	22
FoxO signaling pathway	map04068	12	6	13	6	10	5	14	7

Osteoclast differentiation	map04380	6	9	7	9	7	12	7	12
PI3K-Akt signaling pathway	map04151	17	15	15	19	12	16	18	18
Transcriptional misregulation in cancer	map05202	20	5	19	10	24	11	24	13
Inflammatory bowel disease (IBD)	map05321	3	6	2	9	2	11	5	9
Salmonella infection	map05132	3	11	2	13	3	13	3	13
Hippo signaling pathway	map04390	9	8	13	9	13	9	17	9
Human papillomavirus infection	map05165	12	14	14	16	17	12	21	11
Basal cell carcinoma	map05217	5	3	7	5	8	3	9	3
Breast cancer	map05224	7	7	9	8	11	5	13	5
Toll-like receptor signaling pathway	map04620	5	6	4	8	4	10	5	9
Pathways in cancer	map05200	20	19	22	25	27	22	33	22
