# Supplementary figures and images for: RNA Sequencing Reveals the Upregulation of FOXO Signaling Pathway in Porphyromonas gingivalis Persister-Treated Human Gingival Epithelial Cells
Source: Int J Mol Sci. 2022 May 20;23(10):5728. doi: 10.3390/ijms23105728 (PMC9146424; doi:10.3390/ijms23105728)

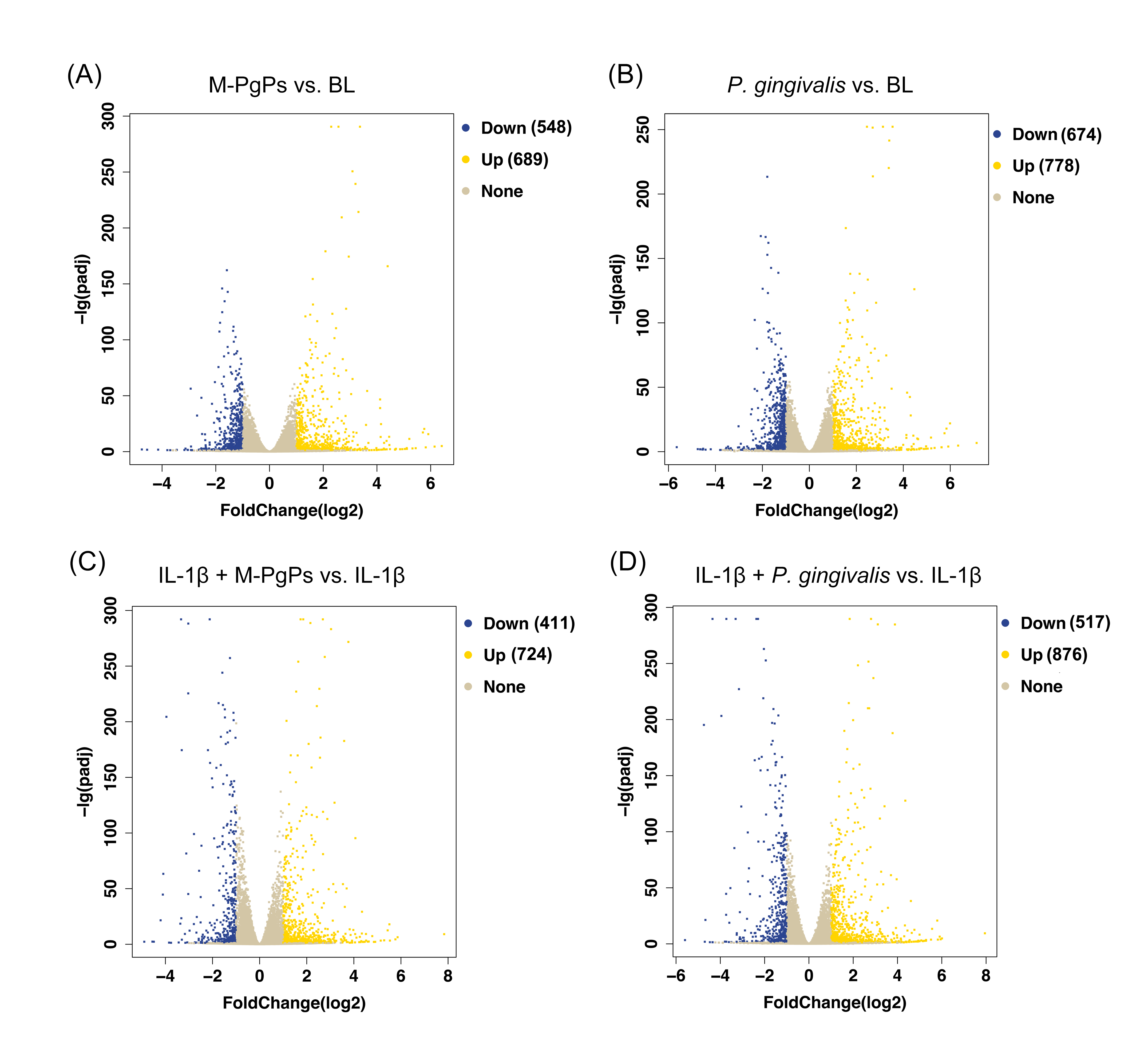

Supplement: Supplementary file 1 [file ijms-23-05728-s001.zip › Supplementary files/Figure S1.tif]

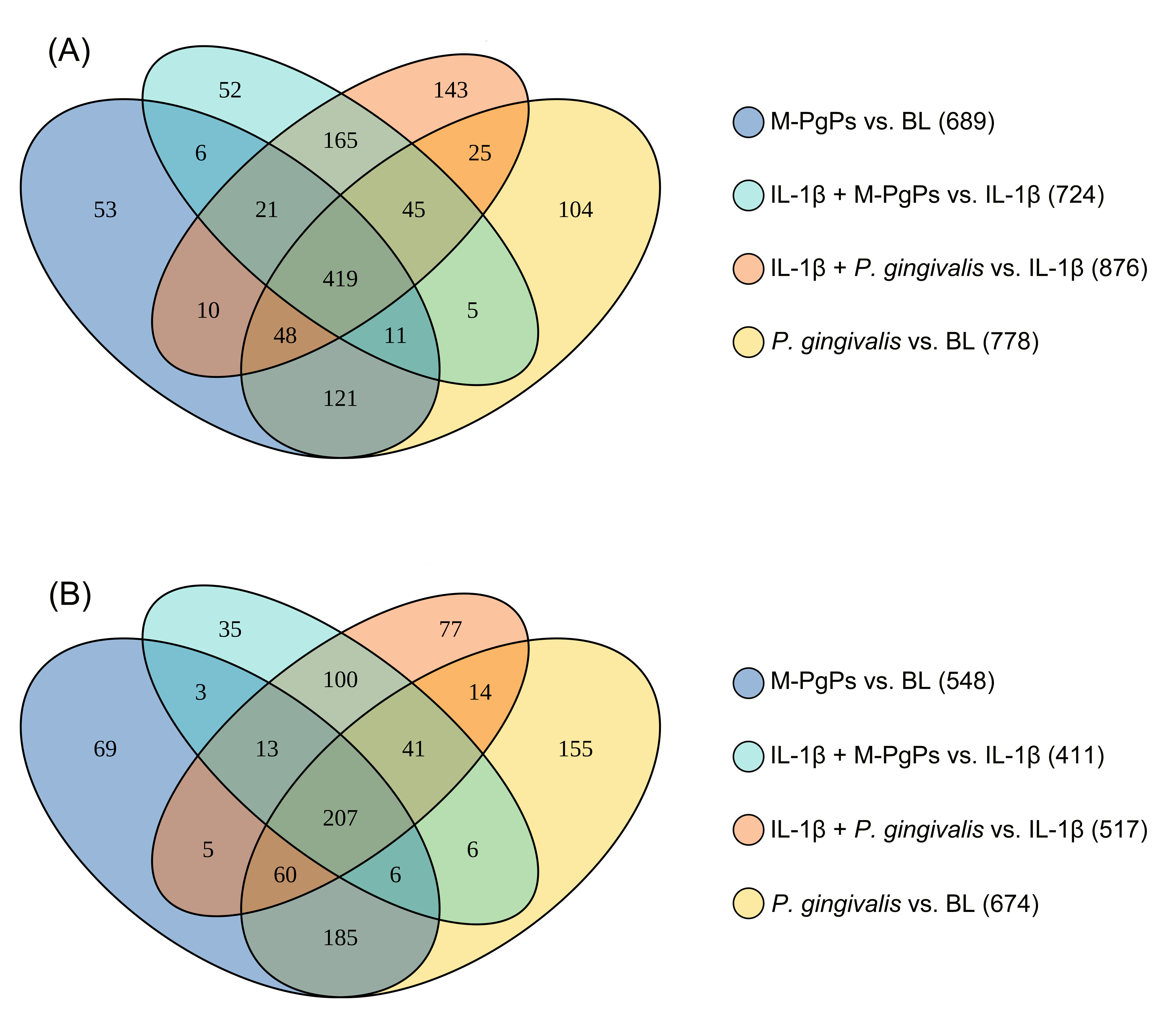

Supplement: Supplementary file 1 [file ijms-23-05728-s001.zip › Supplementary files/Figure S2.tif]

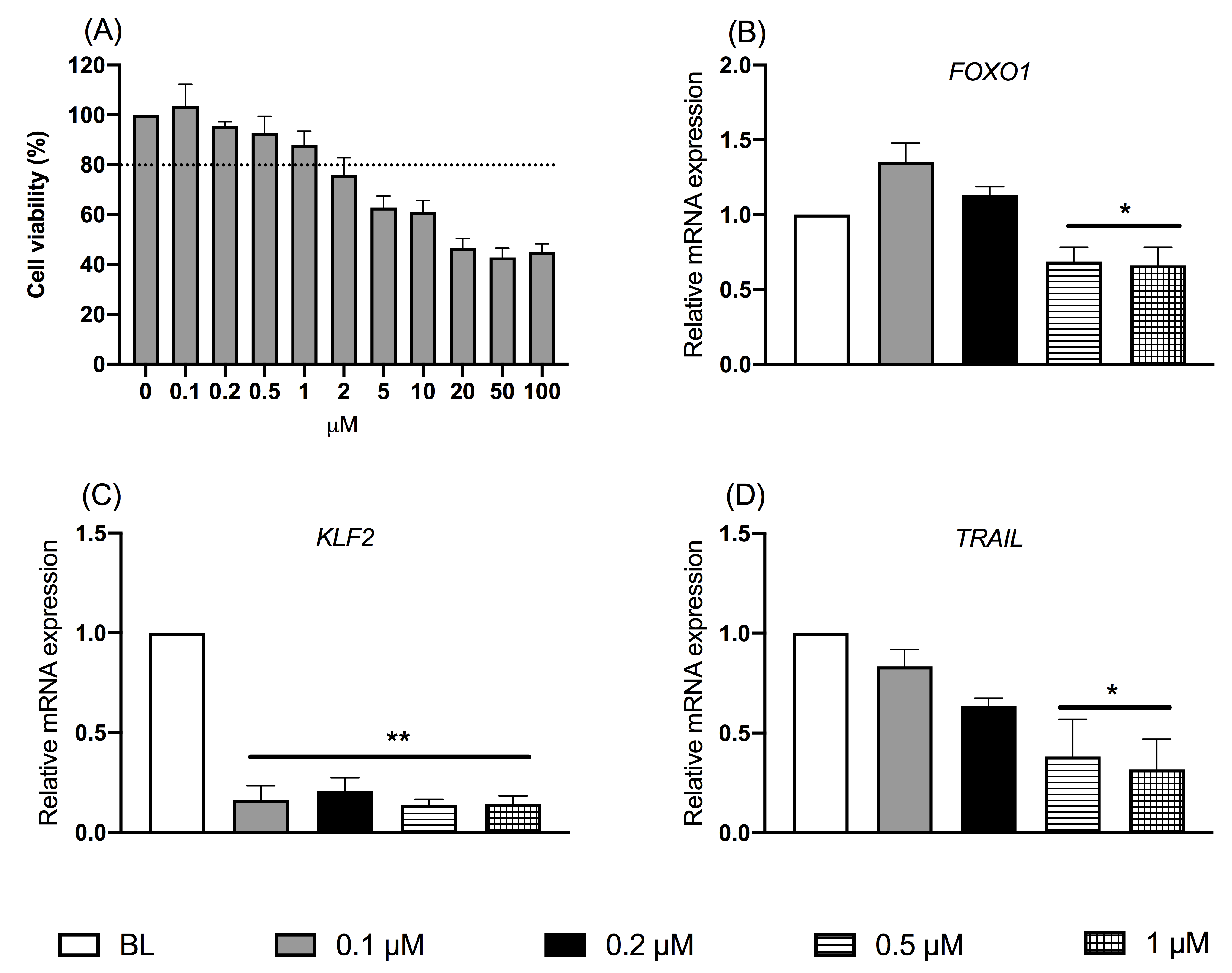

Supplement: Supplementary file 1 [file ijms-23-05728-s001.zip › Supplementary files/Figure S3.tiff]

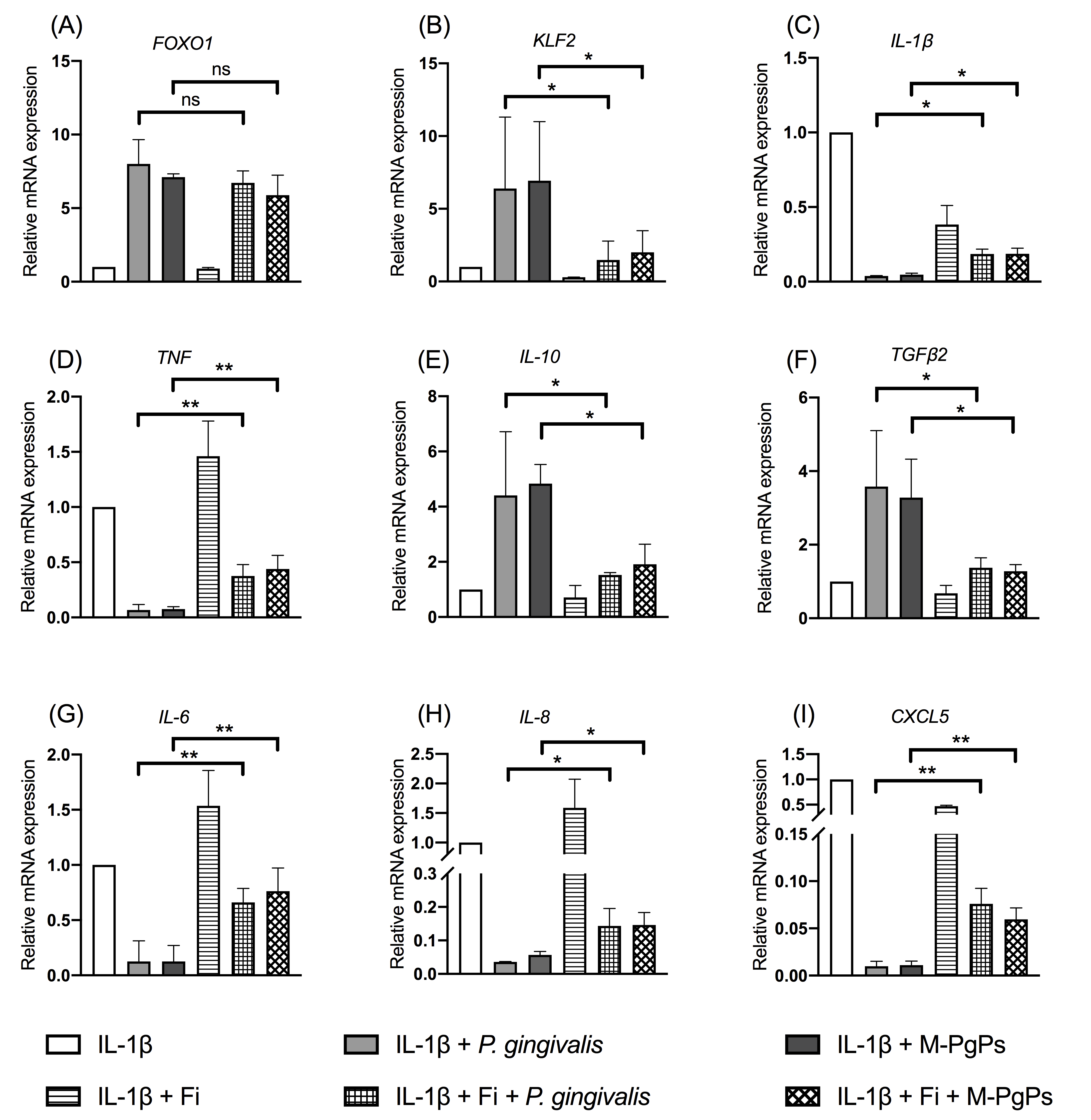

Supplement: Supplementary file 1 [file ijms-23-05728-s001.zip › Supplementary files/Figure S4.tiff]
